# Sodium valproate, a potential repurposed treatment for the neurodegeneration in Wolfram syndrome (TREATWOLFRAM): trial protocol for a pivotal multicentre, randomised double-blind controlled trial

**DOI:** 10.1136/bmjopen-2024-091495

**Published:** 2025-02-26

**Authors:** Renuka P Dias, Kristian Brock, Kun Hu, Rajat Gupta, Una Martin, Andrew Peet, Martin Wilson, Patrick Yu-Wai-Man, Benjamin Wright, Susan Mollan, Archana Kulkarni, Isabelle Meunier, Lucinda Billingham, Zsuzsanna Nagy, Heather Rose, Sulev Koks, Malgorzata Zatyka, Dewi Astuti, Tracy Lynch, Karen E Morrison, Darren Barton, Sabrina Cronier, Rebecca Malpass, Ruth Evans, Amandip Malhi, Pooja Takhar, Amy Lamb, Gema Esteban-Bueno, Wojciech Młynarski, Christophe Orssaud, Agathe Roubertie, Victoria Homer, Timothy Barrett

**Affiliations:** 1 Institute of Applied Health Research, University of Birmingham, Birmingham, UK; 2 Department of Paediatric Endocrinology, Birmingham Women's and Children's Hospital, Birmingham, UK; 3 Cancer Research UK Clinical Trials Unit, University of Birmingham College of Medical and Dental Sciences, Birmingham, UK; 4 Department of Cancer and Genomic Sciences, University of Birmingham, Birmingham, UK; 5 Department of Paediatric Neurology, Birmingham Women's and Children’s NHS Foundation Trust, Birmingham, UK; 6 Institute of Clinical Sciences, University of Birmingham, Birmingham, UK; 7 Paediatric Oncology, Birmingham Women's and Children's Hospital, Birmingham, UK; 8 Centre for Human Brain Health, School of Psychology, University of Birmingham, Birmingham, UK; 9 John van Geest Centre for Brain Repair and MRC Mitochondrial Biology Unit, Department of Clinical Neurosciences, University of Cambridge, Cambridge, UK; 10 Cambridge Eye Unit, Addenbrooke’s Hospital, Cambridge, UK; 11 Moorfields Eye Hospital NHS Foundation Trust, London, UK; 12 Institute of Ophthalmology, University College London, London, UK; 13 Department of Neurology, University Hospitals Birmingham NHS Foundation Trust, Birmingham, UK; 14 Birmingham Neuro-Ophthalmology, Queen Elizabeth Hospital, University Hospitals Birmingham NHS Trust, Birmingham, UK; 15 Department of Ophthalmology, Birmingham Women's and Children’s NHS Foundation Trust, Birmingham, UK; 16 National reference centre for inherited retinal dystrophies, University hospital of Montpellier, Montpellier, France; 17 Institute of Neurosciences Inserm, Montpellier University, Montpellier, France; 18 Department of Inflammation and Aging, University of Birmingham, Birmingham, UK; 19 Medical Physics and Clinical Engineering, Nottingham University Hospitals NHS Trust, Nottingham, UK; 20 Centre for Molecular Medicine and Innovative Therapeutics, Murdoch University, Perth, Western Australia, Australia; 21 Wolfram Syndrome UK, Worthing, UK; 22 School of Medicine, Dentistry and Biomedical Sciences, Queen’s University Belfast, Belfast, UK; 23 UGC Almeria Periferia, Distrito Sanitario Almeria, Sistema Sanitario Publico Andaluz (SSPA), Consejeria de Salud y Consumo (Junta de Andalucia), Seville, Spain; 24 Department of Paediatrics, Oncology and Haematology, Medical University of Lodz, Lodz, Poland; 25 Functional Unit of Ophthalmology; Ophthalmological Rare Diseases Reference Center, Functional Unit of Ophthalmology; Ophthalmological Rare Diseases Reference Center, Paris, France; 26 Département de Neurologie Pédiatrique, CIC pédiatrique, CHU Gui de Chauliac, INM, INSERM U 1298, Montpellier, France; 27 Department of Paediatric Endocrinology, Birmingham Women's and Children’s NHS Foundation Trust, Birmingham, Birmingham, UK

**Keywords:** Drug Therapy, Neuro-ophthalmology, Randomised Controlled Trial, Clinical trials

## Abstract

**Introduction:**

Wolfram syndrome (*WFS1-*Spectrum Disorder) is an ultra-rare monogenic form of progressive neurodegeneration and diabetes mellitus. In common with most rare diseases, there are no therapies to slow or stop disease progression. Sodium valproate, an anticonvulsant with neuroprotective properties, is anticipated to mediate its effect via alteration of cell cycle kinetics, increases in p21^cip1^ expression levels and reduction in apoptosis and increase in Wolframin protein expression. To date, there have been no multicentre randomised controlled trials investigating the efficacy of treatments for neurodegeneration in patients with Wolfram syndrome.

**Methods and analysis:**

TREATWOLFRAM is an international, multicentre, double-blind, placebo-controlled, randomised clinical trial designed to investigate whether 36-month treatment with up to 40 mg/kg/day of sodium valproate will slow the rate of loss of visual acuity as a biomarker for neurodegeneration in patients with Wolfram syndrome. Patients who satisfied the eligibility criteria were randomly assigned (2:1) to receive two times per day oral gastro-resistant sodium valproate tablets up to a maximum dose of 800 mg 12 hourly or sodium valproate-matched placebo. Using hierarchical repeated measures analyses with a 5% significance level, 80% power and accounting for an estimated 15% missing data rate, a sample size of 70 was set. The primary outcome measure, visual acuity, will be centrally reviewed and analysed on an intention-to-treat population.

**Ethics and dissemination:**

The protocol was approved by the National Research Ethics Service (West of Scotland; 18/WS/0020) and by the Medicines and Healthcare products Regulatory Agency. Recruitment into TREATWOLFRAM started in January 2019 and ended in November 2021. The treatment follow-up of TREATWOLFRAM participants is ongoing and due to finish in November 2024. Updates on trial progress are disseminated via Wolfram Syndrome UK quarterly newsletters and at family conferences for patient support groups. The findings of this trial will be disseminated through peer-reviewed publications and international presentations.

**Trial registration number:**

NCT03717909.

**STRENGTHS AND LIMITATIONS OF THIS STUDY**

- This study has been designed as a ‘gold standard’ double-masked international multicentre randomised controlled trial, to provide robust evidence for the effectiveness or otherwise of the intervention.
- The Pivotal Trial design allowed incorporation of all data required for a future marketing authorisation application should the trial demonstrate efficacy.
- Patient support groups from four countries chose the primary outcome measure (visual acuity) as the highest priority for affected people and their carers.
- The trial does not include children under 6 years, or adults for whom vision has already been lost.

## Introduction

 Wolfram syndrome is a rare combination of neurodegeneration and diabetes, with an estimated prevalence of 1:500 000.^1^ The minimum clinical diagnostic criteria include both diabetes mellitus and progressive optic atrophy in childhood.^2^ It shows primarily autosomal recessive inheritance, due to mutations in the gene *WFS1*.^3 4^ Approximately 50% of affected children develop signs of neurodegeneration such as cerebellar ataxia and brainstem function impairment by 15 years of age^5^; and severe vision impairment by 18 years.^6^ Up to two-thirds of affected people develop cranial diabetes insipidus, sensorineural deafness and neurogenic bladder; other complications include hypogonadism in males, neurological and psychiatric disorders.^2^ There is currently no specific treatment for this progressive disorder.

The protein*,* Wolframin, negatively regulates endoplasmic reticulum (ER) stress through suppression of ATF6 signalling,^7^ maintenance of ER calcium homeostasis,^8^ cell cycle progression^9^ and granular acidification.^10^ Depletion of Wolframin expression and/or activity results in cell death. A potent cyclin-dependent kinase inhibitor, p21^cip1^, functions as a regulator of cell cycle progression at the G1 and S phase.^11^ It inhibits the apoptosis induced by cell cycle arrest and p53 expression. We observed significant p21^cip1^ downregulation in three Wolframin-depleted cell lines at baseline; however, the expression of p21^cip1^ was associated with inhibition of progression through the G2 phase of the cell cycle and prevention of neuronal apoptosis.^12^

p21^cip1^ has been proposed as a potential therapeutic agent that produces functional regeneration following central nervous system injuries.^13^ It is also known to induce/promote synaptogenesis and the survival of new neurons.^14 15^ Its expression can be increased by known drugs (high content screen of 1040 US Food and Drug Administration (FDA)-approved drugs carried out by Nagy *et al*, University of Birmingham; unpublished). Based on these findings, a panel of 22 drugs was screened that are known to increase expression of p21^cip1^, that cross the blood-brain barrier and that have a reasonable side effect profile. Sodium valproate (VPA) was selected based on: a well-established safety profile; decades of use as a licensed medicine in children; freedom to operate; and European Medicines Agency (EMA) (EU/3/14/1428) and FDA (08/05/2015) Orphan Drug Designation 2015: sodium VPA for the treatment of Wolfram syndrome; Core Technology Patent WO2014049366).

VPA is currently approved for the treatment of epilepsy and bipolar disorder and potentiates the inhibitory action of gamma amino-butyric acid (GABA) through an action on the further synthesis or further metabolism of GABA.

Our hypothesis is that VPA-induced p21^cip1^ expression may prevent neuronal death through its ability to halt re-entry of neurons into the cell cycle, or arrest progression through the G1/S checkpoint, allowing neuronal survival. Our aim is that treatment with VPA will slow the progression of neurodegeneration in patients with Wolfram syndrome.

## Methods

### Patient and public involvement

60 people with Wolfram syndrome were consulted from Wolfram syndrome UK,^16^ French Wolfram Association, Spanish Association for Research and Support for Wolfram Association and the Snow Foundation (USA). They were asked to rank symptoms in order of importance, and the highest ranked symptom was vision loss. In addition to being a patient-relevant outcome measure, natural history data in visual acuity (VA) was available; VA showed linear progression over time; there was a standardised and reproducible method of measurement; and it was possible to take repeated measures over time. We therefore designed a trial with the rate of vision loss as the primary outcome. The decline of VA over time is associated with optic nerve atrophy,^17^ thus making it a biomarker of disease activity in Wolfram syndrome. The CEO of the UK charity Wolfram Syndrome UK (WSUK; TL) is a member of TREATWOLFRAM’s Trial Steering Committee (TSC).

### Trial design overview

**Figure 1:**
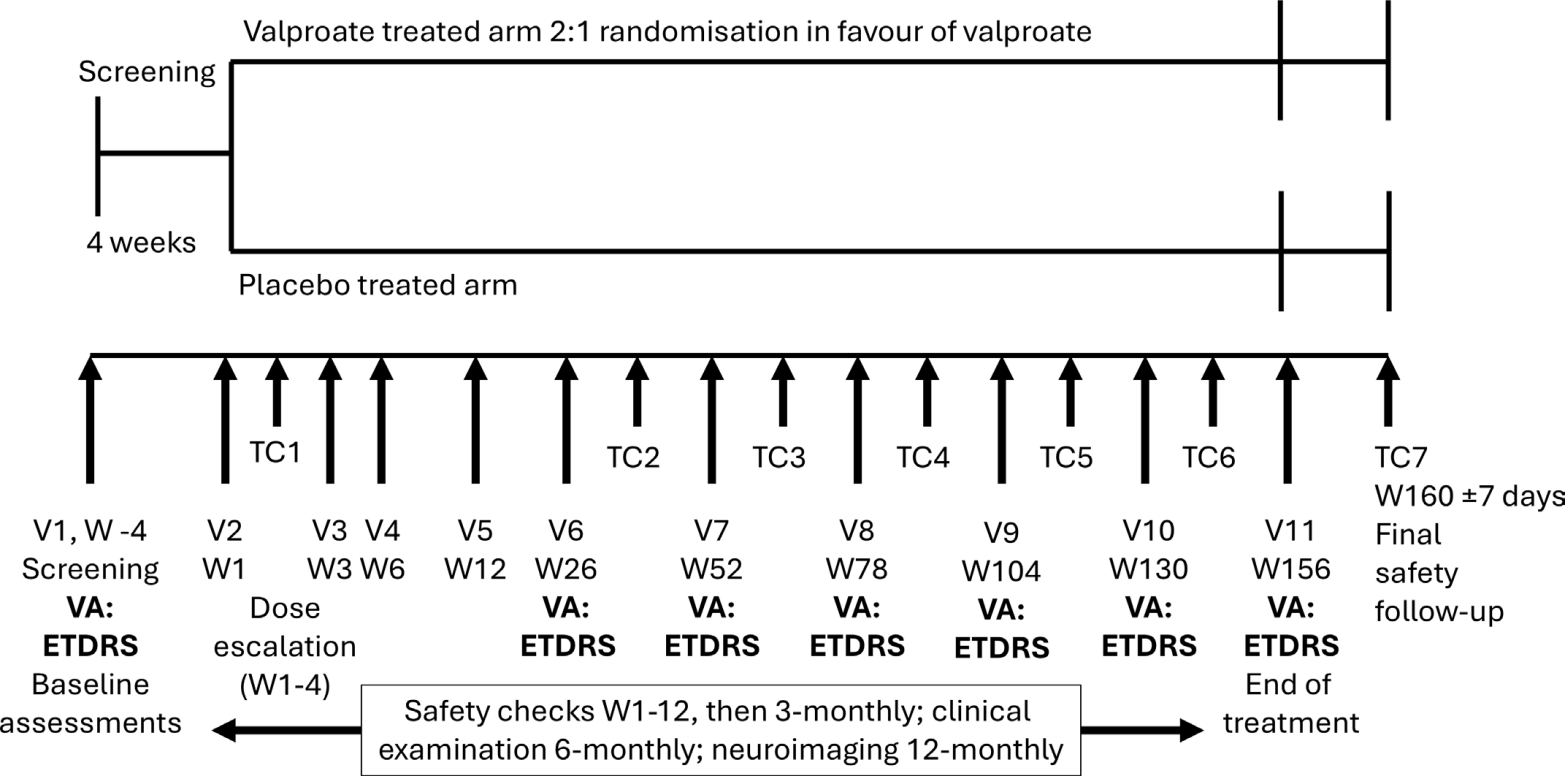
Schematic of sodium valproate efficacy and safety in Wolfram syndrome trial design. Eligible participants are randomly assigned to a 36-month treatment of once-daily or twice-daily sodium valproate or placebo control. Both the trial investigators and the participants are blinded to drug allocation. ETDRS, early treatment diabetic retinopathy study chart; TC, telephone call; V, visit; VA, visual acuity assessment; W, week.

TREATWOLFRAM is a multicentre, double-blind, placebo-controlled randomised clinical trial of treatment for 36 months with VPA as 200 mg gastro-resistant tablets, for children and adults with Wolfram syndrome confirmed by at least one pathogenic mutation in the Wolfram gene. The primary aim is to determine the efficacy of VPA compared with placebo in slowing the rate of deterioration of VA. Eligible participants are randomly assigned by the clinical trials unit to once-daily or twice-daily treatment with either VPA or VPA-matched placebo (control) in a 2:1 ratio (VPA:placebo). A schematic of the trial design is summarised in figure 1.

The Standard Protocol Items: Recommendations for Intervention Trials checklist^18^ is provided as online supplemental appendix 1 and WHO Trial Registration Data Set in online supplemental appendix 2.

### Patient selection

Eligible children and adults (6 years old and above) are identified and invited to participate at the participating trial site centres in the UK (Queen Elizabeth University Hospital, Birmingham Women’s and Children’s Hospital), and internationally, Servicio Andaluz de Salud de la Consejería de Salud y Familias in Almeria, Spain; Medical University of Lodz, Poland; Hôpital Européen Georges Pompidou and Assistance Publique Hôpitaux de Paris, France; and CHU Gui de Chauliac, Montpellier, France.

### Consent

It is the responsibility of the local principal investigator to obtain written informed consent from each participant, and for paediatric participants, obtain the participant’s parent/legal guardian consent, with every reasonable effort to obtain a child’s assent, prior to performing any trial-related procedure. Age-specific participant information sheets and parent/legal guardian information sheets are available from the trials office (treatwolfram@trials.bham.ac.uk) as well as age-specific and parent/guardian-specific informed consent forms. Example adult participant information sheets and consent forms are included in onlinesupplemental appendices 3  4, respectively.

### Participant pathway

The TREATWOLFRAM trial involves 11 participant-related visits at their nearest trial site as follows: (1) screening, enrolment, baseline investigations and randomisation (visit 1); (2) dose escalation and safety visits (visits 2, 3); (3) dose maintenance (visits 4–11, up to 156 weeks post randomisation); and (4) post-treatment follow-up (telephone call 4 weeks after cessation of treatment). The schedule of trial visits and data collection is summarised in online supplemental appendix 5.

### Treatment groups

#### Sodium valproate (active experimental group)

Sodium VPA 200 mg gastro-resistant tablets, containing the active substance VPA, ATC code N03AG01, is supplied by Wockhardt UK. This is packaged and labelled in accordance with EMA regulations on Good Manufacturing Practice.

#### Sodium valproate-matched placebo (inactive, placebo-control group)

Placebo is packaged, labelled and distributed by a third party mirroring that of VPA. Placebo is identical in appearance to VPA 200 mg gastro-resistant tablets.

### Dose modifications

VPA treatment commences at a dose based on the participant’s body weight (10 mg/kg/day in one or two divided doses) and increases in 200 mg or 400 mg increments over 4 weeks to 40 mg/kg/day or a maximum of 800 mg/day for participants aged between 6 years and less than 12 years, or a maximum of 1600 mg/day for those aged 12 and over. For daily doses of 400 mg or more, the doses are given as two divided doses 12 hours apart. Participants then remain on this dose until the end of the trial, unless adverse events are experienced.

In the event of a moderate adverse event (AE) that is possibly related to the Investigational Medicinal Product (IMP), participants are assessed and their daily dose of IMP reduced by 200 mg increments or more per week for adults until tolerated; or by 10 mg/kg increments per week for children. Participants are then asked to continue on the lower dose to the end of the trial unless, in the opinion of the local investigator, a higher dose is likely to be tolerated. If a participant has temporarily discontinued treatment, they may also be allowed to restart treatment, provided that:

- The participant stopped IMP for less than 180 days (both continuously and cumulatively).
- The AE(s) that prompted the dose reduction or cessation have resolved.
- If the AEs included abnormal blood investigations such as liver function tests, these have returned to the normal range.

### Concomitant therapy

All concomitant medications given in relation to standard clinical management are recorded for this trial via the electronic case report form database. Within the summary of product characteristics (SmPC) is an extensive list of drugs that can interact with VPA. Prohibited concomitant medications and those to be used with caution are listed in online supplemental appendix 6.

In addition to trial medications, participants continue to receive standard national care recommendations concerning diabetes mellitus (ie, glucose-lowering agents) and other coexisting illnesses throughout the trial. All participants follow their national pregnancy prevention programmes for patients taking VPA. The use of VPA in pregnancy is associated with a teratogenic risk to the fetus. Therefore, an inclusion criterion was that all women of childbearing potential must use a highly effective method of contraception, without interruption, for the whole of the IMP period.

### Treatment compliance

Treatment compliance is monitored by a review of the numbers of returned tablets in the used tablet containers and the participants’ self-completed treatment medication diaries. The latter provide written evidence of dosage, time and date when each participant administers the study drug. For all participants, plasma VPA levels are measured at baseline, day 42, day 360, day 720 and day 1080 in an independent central laboratory in Birmingham (UK). Results are provided every 6 months to the trial statistician, who then sends a report to the Data Monitoring Committee.

### Outcome measures

#### Primary outcome measure

The primary outcome measure is the rate of change in VA as assessed by corrected VA in each eye, measured on the Logarithm of the Minimum Angle of Resolution (LogMAR) scale using Early Treatment Diabetic Retinopathy Study (ETDRS) charts. The VA is calculated by testing each eye independently using the patient’s own refractive correction (glasses or contact lenses), as required. Increases in LogMAR represent deterioration. Due to the potential for assessor error, completed ETDRS charts are sent to an independent central reviewer who reviews calculations. VA is assessed at screening, and then at day 0 (baseline), 180, 360, 540, 720, 900 and 1080.

#### Secondary outcome measures

- Safety, measured by adverse events according to the National Cancer Institute’s (NCI) Common Terminology Criteria for Adverse Events (CTCAE) V.4.0.^19^
- Tolerability, measured by dose achieved, days of treatment and treatment-related dose reductions and discontinuations.
- Pons volume (PV), a surrogate marker for neurodegeneration, measured in mm^3^, estimated from standardised analysis of MRI of the brain.
- Brainstem and brainstem substructure volumes, measured by MRI.
- Peripapillary retinal nerve fibre thickness, measured by optical coherence tomography.
- Colour vision measured by Hardy Rand and Rittler test of Isihara plates.^20^
- Contrast sensitivity, if available.
- Comprehensive ophthalmic examinations included slit-lamp assessment of the anterior and posterior segment, documentation of pupil examination and eye movement examination including strabismus and nystagmus.
- Sleep, measured by questionnaires (described in the Questionnaires subsection).
- Balance, measured by Mini-Balance Evaluation Systems Test (Mini-BESTest).^21^
- Hearing, measured by pure tone audiometry.
- Wolfram Unified Rating Scale.^22^
- Mood, measured by the KIDSCREEN questionnaire.^23^
- Quality of life, measured by questionnaires (described in the section below).
- Pancreatic beta cell reserve, measured by mixed meal tolerance test or equivalent (UK participants only, optional).

### Analytical methods

#### Brain imaging

MRI assessments are conducted at screening (day −28); month 12 visit (day 360); month 24 visit (day 720); and month 36 visit (day 1080). While there is some flexibility between centres, all longitudinal scan sequences on an individual participant must be identical with regards to head coil choice, scanner make and model, field strength and scan parameters.

The T1-weighted volume scan is segmented centrally by the imaging team to estimate the volume of the whole brain stem, brainstem substructures and specifically PV. Global and regional brain volumes will also be measured to disambiguate any brainstem changes with global growth or atrophy. The T2-weighted volume scan sequence enables clinical reporting of the scans to assess the safety of VPA in participants. Additionally, the T2-weighted volume assists in the calculation of total intracranial volume, needed to normalise the brainstem and PV measurements.

#### Clinical and laboratory data

Biochemistry assessments include blood (serum or plasma) urea, creatinine, electrolytes (sodium, potassium), bone chemistry (plasma calcium, phosphate, alkaline phosphatase, vitamin D, parathyroid hormone), lactate, random glucose, random plasma osmolarity and urine osmolarity, glycated haemoglobin, liver function tests (total bilirubin, alanine transaminase, aspartate transaminase, gamma glutamyl transferase), coagulation (prothrombin time, thrombin time, activated partial thromboplastin time, international normalised ratio, fibrinogen), ammonia, amylase. Haematology assessments include haemoglobin, white count and differential, platelet count. Thyroid function tests (TFT) will include thyroid-stimulating hormone, free triiodothyronine and free thyroxine. TFT are performed at baseline (visit 1), day 90 (visit 5), day 360 (visit 7), day 720 (visit 9) and day 1080 (visit 11).

#### Questionnaires

The following questionnaires are used in this trial:

- International Consultation on Incontinence Questionnaires (ICIQ): ICIQ Female Lower Urinary Tract Symptoms,^24^ ICIQ Male Lower Tract Symptoms^25^ and ICIQ Paediatric Lower Urinary Tract Symptoms (CLUTS both self-administration and caregiver reported).^26^
- Quality of life questionnaires: Pediatric Quality of Life Inventory (child 8–12, parent of child 8–12, teen 13–18, parent of teen 13–18).^27^
- Sleep questionnaires: Sleep-related Breathing Disorder scale extracted from the Paediatric Sleep Questionnaire (referred to as PSQ) parent report for those under 18^28^ and Pittsburgh Sleep Quality Index self-report.^29^
- Visual function questionnaires: Vision-related Quality of Life for Children (for patients aged 8–12 years), Vision-related Quality of Life for Young People (for patients aged 12–18 years),^30^ Functional Vision Questionnaire for Children (for patients aged between 8 and 12 years), Functional Vision Questionnaire for Young People (for patients aged 13–18 years)^31^ and the 25-item Visual Function Questionnaire (both self-report or interviewer-administered for adults).^32^
- Mood questionnaires: KIDSCREEN (patients aged 8–18)^33^ and Hospital Anxiety and Depression Score (adults).^34^

For non-UK sites, questionnaires were administered only if there was a validated translated version available in the country.

### Statistical analysis

#### Sample size

The target sample size for this trial is pragmatic, driven by what is required to conduct a rigorous scientific trial and what is feasible. Using hierarchical repeated measures analyses with a 5% significance level, 80% power and accounting for an estimated 15% missing data rate, the total sample size is 70, to be recruited over all sites with no one site recruiting more than 50% of all participants. The methods of Diggle *et al* were used for the sample size calculation.^35^

A longitudinal hierarchical model analysis for VA has been chosen because: (1) Wolfram syndrome symptoms progress gradually over years; (2) a repeated measures analysis yields a feasible sample size.

We used data on a cohort of 26 patients with Wolfram syndrome, kindly provided by Professor Tamara Hershey (St Louis Wolfram Research Clinic, USA). Our calculation assumed:

- Mean progression of VA loss under placebo patients was 0.082 LogMAR units per annum (p.a.).
- The effect of being on active treatment resulting in an effect size of 0.041 p.a. (representing a halving of the rate of progression).
- Homoscedastic errors distributed N (0, 0.074^2^).
- Random gradients N (0, $\sigma_{B}$). In the St Louis cohort data, we noted that a rapidly progressing patient inflated the estimate of $\sigma_{B}$. This patient was measured using off-chart methods, which will not be used in the primary analysis of TREATWOLFRAM. Initially, we estimated $\sigma_{B}$ to be 0.036, the estimate obtained by fitting the model to the St Louis data, excluding the patient with off-chart measurements. We assessed sensitivity to this figure (by including observations from this patient) in our simulation study.
- That VA LogMAR scores at baseline range from 0 to 1.6 to reflect the patients that will be recruited in the trial (VA greater than 1.6 is an exclusion criterion).

We expect that some participants will discontinue early, leading to less than 100% data collection for the primary outcome. We assume that participants provide data until they discontinue at some random point, with all subsequent observations missing. Simulations show that this method of incorporating missing data is conservative and that a target sample size of 70 participants when accounting for up to 15% data missing has a type I error sufficiently close to 5% and power approximately equal to 80%.

Finally, the analysis presented above uses patient-level series. We expect power to increase in our trial analysis because we will analyse series at the eye level. Analysing by eye yields approximately double the number of series. However, the series within patients will be highly correlated, so the effective sample size will be less than double the number of participants.^36^

#### Primary outcome analysis

The primary outcome will be analysed according to the intention-to-treat principle and longitudinal analysis of patients’ progression of VA loss between evaluations at baseline and after 36 months of treatment measured.

We expect VA loss to progress linearly in time for both placebo and VPA-treated participants, on average, although at different rates. We depart from the analysis above by proposing to analyse the series in eyes separately, corrected with glasses, nested within patient. Thus, we initially propose random intercepts for individuals and for eyes within individuals. The patient-level intercept will address the tendency for the vision in the eyes of individual patients to be highly correlated. Additionally, we expect random gradients to be necessary at the eye level to allow that each eye may have its own rate of progression.

Our proposed initial model to analyse VA is:

$$y_{ijk}=\left(\alpha +a_{i}+\;a_{ik}\right)+\left(\beta +\gamma z_{j}+b_{ik}\right)t_{j}+\;\epsilon_{ijk},\;\;i=1,\ldots ,N,\;j=0,\ldots ,6,\;k=0,1$$

Let *i* index patients, so that *i*=1, …, *N*, where *N* is the total trial sample size. Let *t*=*t_VA_* and *j* index *t*, so that *t_0_*=0 and *t_6_*=3 years, etc. Finally, let *k*=0 denote the left eye, and *k*=1 denote the right eye. Let *y_ijk_* denote VA for patient *i* at time *t_j_* in eye *k*. Let *z_i_* take the value 1 if patient *i* is allocated to VPA, else 0.

This model will be fitted in R using the packages nlme or lme4, using restricted maximum-likelihood (REML). General positive-definite covariance structure will be used.

This model assumes that: the patient random intercepts $a_{i}$ are independent for different *i*; the eye random intercepts $a_{ik}$ and slopes $b_{ik}$ are independent for different *i* and *k;* and the within-group errors $\epsilon_{ijk}$ are independent for different *i*, *j* and *k* and independent of the random effects.

Here, α is the fitted mean VA value at *t*=0, β is the mean progression in VA per annum for participants on placebo and γ is the adjustment to the mean progression in VA for participants on VPA, a term we will refer to as the treatment effect.

The sign of γ tells us about the direction of the treatment effect. Negative values represent benefit. The presence or absence of a treatment effect that is unlikely to have occurred through random variation alone will be tested via t-test of the γ-coefficient in the REML fit of the hierarchical model, as recommended by Pinheiro and Bates.^37^

If this model does not fit the observed data satisfactorily, alternative models will be considered.

### Randomisation

Eligible participants were randomly assigned on a 2:1 basis in favour of VPA to either of the two trial treatments (VPA or placebo) by the local site investigators using computer-generated randomisation at the Cancer Research UK Clinical Trials Unit (CRCTU). Trial participants were allocated a unique trial identification number via a list generated by the unblinded trial statistician using a block size of between 6 and 12. To ensure blinding was maintained, treatment allocation was not displayed; rather, pack numbers to be dispensed were shown.

### Masking

Both VPA and placebo control are distributed by a third party ensuring the receiving trial site is masked to the drug throughout the duration of the trial, that is, trial investigators and pharmacists, outcome assessors, participants and their carers are masked from the drug allocation. Only data analysts remain unblinded, that is, the trial statistician and the database programming team.

A 24-hour, 7 days a week unblinding service is provided by Emergency Scientific and Medical Services Global. The unblinding service will allow the local principal investigator, or other medically qualified person, to identify the study medication (sodium VPA or matched placebo) for an individual patient in an emergency situation.

At the end of the trial, unblinded allocations will be released to each site for feedback to the trial participants.

### AE reporting and analysis

Participants are monitored regularly for AEs (definitions are listed in online supplemental appendix 7).

The reporting period for AEs starts at the screening visit and continues until 30 days after the last IMP administration. Serious adverse events (SAEs) are reported until 30 days post end of treatment. The NCI CTCAE^19^ are used to grade each AE. The trial office records all AEs reported (nature, onset, duration, severity, outcome) evaluates them with respect to seriousness, and site-assessed causality and expectedness. Interim analysis of SAEs is performed and presented to the independent Data Management Committee (DMC) regularly.

Specific attention is given to AEs related to liver and renal function considering previous safety data relating to VPA-induced hepatic encephalopathy and interstitial nephritis. VPA is also known to have a high teratogenic potential, and children exposed in utero to VPA have a high risk for congenital malformations and neurodevelopmental disorders. We therefore monitor the outcome of pregnancies of trial participants to provide SAE data on congenital anomalies or birth defects.

### Storage of trial samples and images

Glucose is analysed at study sites. C-peptide and pro-insulin are analysed by the Core Biochemical Assay Laboratory (Cambridge, UK). VPA levels are analysed at the Regional Toxicology Laboratory, Sandwell General Hospital (UK).

MRI scans are anonymised using a Conformité Européene (CE) mark anonymisation tool at the local site, before transfer to the Clinical Trials Unit. The staff at the local centre acquiring the MRI then transfer anonymised data to a compact disc, encrypted where possible and delivered by courier to the trials office, where they are transferred to the central imaging server.

Optional consent in UK participants only is requested for:

- Health records are to be flagged through the NHS Information Centre for Health and Social Care service to assist with long-term follow-up data collection.
- Any remaining research blood samples being stored and used for future research purposes.
- The collection, storage and analysis of skin samples for future research.

### Data handling, quality assurance and retention

Data management is undertaken by the CRCTU at the University of Birmingham, UK. The CRCTU is fully compliant with the Data Protection Act 1998, the International Conference on Harmonisation Good Clinical Practice (GCP) and General Data Protection Regulation (GDPR). Participant identifiable data are shared only with the clinical team on a need-to-know basis to provide clinical care and to ensure appropriate follow-up. All trial records will be archived and securely retained for at least 25 years. On-site monitoring will be carried out as required following a risk assessment and as documented in the Quality Management Plan. Further information regarding data management is provided in the study protocol.

The CRCTU will hold the final trial data set and will be responsible for sharing clinical trial data with the wider research community to maximise potential patient benefit while protecting the privacy and confidentiality of trial participants. Data anonymised in compliance with the Information Commissioners Office requirements, using a procedure based on guidelines from the Medical Research Council Methodology Hubs, will be available for sharing with researchers outside of the trials team within 12 months of the primary publication.

### Trial organisation structure

The University of Birmingham acts as the sponsor of the trial (University of Birmingham, Edgbaston, Birmingham B15 2TT. Email: researchgovernance@contacts.bham.ac.uk).

The Trial Management Group (TMG) is composed of the chief investigator, co-investigators representatives from each National Coordinating Centre and the trial team at the CRCTU. The TMG is responsible for the day-to-day running and management of the trial.

The TSC provides overall supervision for the trial on behalf of the sponsor and advice through its independent chair. Membership includes independent clinicians, the chief investigator, a patient/parent representative and members of the TMG as appropriate.

Data analyses are supplied in confidence to the independent DMC, operating in accordance with a trial-specific charter based on the template created by the DAMOCLES group and consisting of an independent adult physician chair, statistician and senior paediatrician.

### Trial status

Recruitment into the TREATWOLFRAM clinical trial opened in January 2019 and completed in October 2021, with 63 participants (90% of target enrolment) randomised from the six trial sites (University Hospitals Birmingham, UK 11; Birmingham Women’s and Children’s Hospital, UK 14; Almeria, Spain 18; Lodz, Poland 5; Montpellier, France 5; Paris, France 10). This number is seven fewer than planned to allow the trial to complete in a reasonable time. We calculated the minimum number of participants required to achieve 80% power while retaining an acceptable type 1 error. Accepting a type 1 error of 10%, we should still be able to achieve 80% power with 63 participants, while allowing for up to 20% attrition. Online supplemental appendix 8 summarises the recruitment rate. Treatment follow-up and data collection are ongoing, and the last trial visit will take place in November 2024. A summary of the protocol amendments is provided in online supplemental appendix 9.

### Ethics and dissemination

The study will be performed in accordance with the recommendations guiding physicians in biomedical research involving human subjects, adopted by the 18th World Medical Association General Assembly, Helsinki, Finland and stated in the respective participating countries’ laws governing human research and GCP.

The protocol was initially approved on 19 March 2018 by the National Research Ethics Service West of Scotland (REC 1) Committee and the UK Health Research Authority (Ref: 18/WS/0020); the current protocol (V.10.0) was approved on 19 September 2023.

A meeting will be held after the end of the study to allow discussion of the main results among the collaborators prior to publication. The results of this study will be disseminated through national and international presentations and peer-reviewed publications. Manuscripts will be prepared by the TMG and authorship determined by mutual agreement.

The full (detailed) clinical trials protocol is available on request at treatwolfram@trials.bham.ac.uk.

## Discussion

### Trial design

Despite recent advances in non-invasive markers of neurodegeneration (eg, tau variants for Alzheimer’s disease, neurofilament light protein for multiple sclerosis), there are no validated laboratory biomarkers for the assessment of disease progression in Wolfram syndrome. We therefore considered a range of clinical measures that were sufficiently robust to use as a primary outcome measure. VA was chosen on the advice of patient support groups and the EMA protocol assistance service.

The Committee for Medicinal Products for Human Use (CHMP) endorsed the use of a placebo arm in the trial design, given the lack of currently approved or effective treatments for Wolfram syndrome. They agreed with a randomisation in favour of active treatment, to maximise the information for active treatment in this rare condition. The committee also recognised that the wide availability of VPA may represent a risk for the integrity of the trial, and that measuring the blood levels of VPA in patients could be helpful to establish whether such protocol violations have occurred, while still maintaining blinded observations. The CHMP agreed that more than one pivotal study would not be feasible given the rarity of the condition; and that therefore not all the normal requirements for a single pivotal trial (such as statistical evidence stronger than p<0.05) would be required. Finally, they advised using the highest tolerated dose of VPA to maximise the chances of demonstrating an effect.

### Safety profile of sodium valproate

Prior to the start of the TREATWOLFRAM trial, the SmPC for VPA stated special warnings and precautions for use in relation to patients with renal insufficiency, hepatic insufficiency and in women of childbearing potential. In turn, the eligibility criteria (box 1) reflected these warnings by excluding patients with or at risk of such conditions, together with regular biochemical monitoring. In keeping with UK Medicines and Healthcare Regulatory Agency (MHRA) safety measures and EMA recommendations, we followed the VPA pregnancy prevention programme, which aims to minimise the risks that could occur by taking VPA during pregnancy.

Box 1 **Eligibility criteria for TREATWOLFRAM**

**Inclusion criteria**

Patients must meet all of the following criteria to be eligible for enrolment:

- A definitive diagnosis of Wolfram syndrome, as determined by the following:
  - Documented diabetes mellitus diagnosed under 16 completed years according to the WHO or American Diabetes Association criteria and/or documented optic atrophy diagnosed under 16 completed years.

AND

- Documented functionally relevant mutations on one or both alleles of the WFS1 gene based on historical test results (if available) or from a qualified laboratory at screening.

- Aged 6 years or older and weighing at least 20 kg.
- A visual acuity assessed as either the right eye or left eye having a logarithm of the minimum angle of resolution score of 1.6 or better on an Early Treatment Diabetic Retinopathy Study chart, with or without corrected vision.
- Written informed consent (and assent as required).
- Females of childbearing potential will only be included after a negative pregnancy test as per the national valproate pregnancy prevention programme or equivalent. If sexually active, they must agree to use a highly effective contraception measure and to pregnancy testing at each clinical follow-up visit.

Highly effective methods include:

- Combined (oestrogen and progestogen containing) hormonal contraception associated with inhibition of ovulation:
  - Oral.
  - Intravaginal.
  - Transdermal.
- Progestogen-only hormonal contraception associated with inhibition of ovulation:
  - Oral.
  - Injectable.
  - Implantable.
- Intrauterine device.
- Intrauterine hormone-releasing system.
- Bilateral tubal occlusion.
- Vasectomised partner.
- Sexual abstinence.
- Sexually active men with a female partner of childbearing potential must agree to the use of condoms and the use of a highly effective method of contraception by the female partner.
- The patient is willing and able to meet all protocol-defined visits for the duration of the trial.

**Exclusion criteria**

- Clinically significant non-Wolfram-related central nervous system involvement which is judged by the investigator to be likely to interfere with the accurate administration and interpretation of protocol assessments.
- A diagnosis of a mitochondrial myopathy.
- Active liver disease has a personal or family history of liver dysfunction related to known genetic disorders, or the patient has porphyria.
- Received treatment with any investigational drug within the 30 days prior to trial entry.
- Currently taking sodium valproate; or has a known hypersensitivity to sodium valproate or its excipients.
- Any other acute or chronic medical, psychiatric, social situation or laboratory result that, based on the investigator’s judgement, would jeopardise patient safety during trial participation, cause inability to comply with the protocol or affect the trial outcome.
- Currently breastfeeding.
- Known urea cycle disorders.
- Has one of the following disorders: lactose intolerance, the Lapp lactase deficiency or glucose-galactose malabsorption.

### Challenges during the COVID-19 pandemic

The trial was carried out during the COVID-19 pandemic, which made recruitment challenging and increased anxiety for participants. A substantial amendment was approved by the West of Scotland Ethics Committee to permit remote study visits, local delivery of blood investigations and couriering of IMP/placebo to participants’ homes during the COVID-19 pandemic. It was also permitted to have questionnaires completed at home by the participant/parent/carer (as per questionnaire instructions) to minimise time spent in hospital during the visit. There was the potential for an impact on data consistency.

### Future directions for research and clinical practice

This trial design will set the standard for future multicentre pivotal trials in ultra-rare diseases. The work done to ensure MR imaging data compatibility from six international sites will provide a template for other international studies collecting neuroimaging data. Biosamples collected from the UK cohort over the 36 months intervention will support investigations into biomarkers that may be used as surrogate outcome measures to shorten the intervention period in future studies. Standardised natural history data from patients on placebo will be available to support future trial design using prior hypotheses and historical natural history data, thereby reducing participant numbers and intervention duration.

If the trial demonstrates efficacy of VPA in slowing progression of VA loss, then our group will seek an industry partner to prepare marketing authorisation applications for licensing of VPA for the treatment of neurodegeneration in Wolfram syndrome. The clinical data collected will inform clinical teams and patients on clinical management and screening for complications.

### Summary

TREATWOLFRAM is the first international randomised controlled trial in this ultra-rare population. It has been designed as a pivotal clinical trial in children and adults, incorporating all the data required for a future EMA marketing authorisation application. The choice of an already-licensed medicine that is already widely available means that it can be speedily made available to patients if the trial is successful.

## supplementary material

10.1136/bmjopen-2024-091495 online supplemental file 1

10.1136/bmjopen-2024-091495 online supplemental file 2

10.1136/bmjopen-2024-091495 online supplemental file 3

10.1136/bmjopen-2024-091495 online supplemental file 4

10.1136/bmjopen-2024-091495 online supplemental file 5

10.1136/bmjopen-2024-091495 online supplemental file 6

10.1136/bmjopen-2024-091495 online supplemental file 7

10.1136/bmjopen-2024-091495 online supplemental file 8

10.1136/bmjopen-2024-091495 online supplemental file 9
