# Supplementary material for: Sodium valproate, a potential repurposed treatment for the neurodegeneration in Wolfram syndrome (TREATWOLFRAM): trial protocol for a pivotal multicentre, randomised double-blind controlled trial
Source: BMJ Open. 2025 Feb 26;15(2):e091495. doi: 10.1136/bmjopen-2024-091495 (PMC11865774; doi:10.1136/bmjopen-2024-091495)
Supplement: online supplemental file 2 [file bmjopen-15-2-s002.pdf]

Supplementary appendix 2: TREATWOLFRAM World Health Organization Trial Registration Data Set

Data category	Information
Primary registry and trial identifying number	clinicaltrials.gov NCT03717909
Date of registration in primary registry	22-Oct-2018
Secondary identifying numbers	n/a
Source(s) of monetary or material support	Medical Research Council<br>Wolfram Syndrome UK
Primary sponsor	University of Birmingham
Secondary sponsor(s)	n/a
Contact for public queries	Treatwolfram@trials.bham.ac.uk
Contact for scientific queries	Treatwolfram@trials.bham.ac.uk
Public title	Efficacy and Safety Trial of Sodium Valproate, in Paediatric and Adult Patients With Wolfram Syndrome
Scientific title	TREATWOLFRAM: A Pivotal, International, Randomised, Double-blind, Efficacy and Safety trial of Sodium Valproate, in paediatric and adult patients with Wolfram syndrome
Countries of recruitment	UK, Spain, Poland, France

Data category	Information
Health condition(s) or problem(s) studied	Wolfram syndrome
Intervention(s)	Sodium valproate
Key inclusion and exclusion criteria: **Adult Group**	Ages eligible for study: ≥6 years<br>Sexes eligible for study: both<br>Accepts healthy volunteers: no
	Inclusion criteria: Visual acuity assessed as either the right eye or left eye having a LogMAR score of 1.6 or better on an ETDRS chart, with or without corrected vision
	Exclusion criteria: Clinically significant non-Wolfram related central nervous system involvement, mitochondrial myopathy, active liver disease, pregnant or breastfeeding patients
Study type	Interventional
	Allocation: randomised, blinded, placebo-controlled
	Primary purpose: Efficacy
	Phase II
Date of first enrolment	08-Jan-2019
Target sample size	70
Recruitment status	Closed
Primary outcome(s)	Rate of change in visual acuitya s assessed by corrected visual acuity in each eye, measured on the Logarithm of the Minimum Angle of Resolution (LogMAR) scale by sight tests in clinic using EDTRS (Early

Data category	Information
	Treatment Diabetic Retinopathy Study) charts assessed at screening, and then at day 0 (baseline), 180, 360, 540, 720, 900 and 1080.
Key secondary outcome(s)	Safety, measured by adverse events according to the National Cancer Institute's (NCI) Common Terminology Criteria for Adverse Events (CTCAE) version 4.0<br><br>Tolerability, measured by dose achieved, days of treatment, and treatment-related dose reductions and discontinuations<br><br>And, as assessed at screening, and then at day 0 (baseline), 360, 720, and 1080:<br><br>• Pons and brainstem volume, as surrogate markers for neurodegeneration, measured and recorded in mm^3 by standardised analysis by MRI of the Pons or brainstem volumes, respectively<br>• Retinal nerve thickness and visual fields, measured by Optical Coherence Tomography<br>• Colour vision, measured by Hardy Rand and Rittler test<br>• Data on cataracts, afferent pupillary defects, strabismus, nystagmus, fundoscopy and visual evoked potentials if available<br>• Sleep, measured by Sleep-related Breathing disorder scale extracted from the Pediatric Sleep Questionnaire (referred to as PSQ) parent report for those under 18 and Pittsburgh Sleep Quality Index (PSQI) Self-Report questionnaires<br>• Balance, measured by Mini-BESTes<br>• Hearing, measured by pure tone audiometry<br>• Disease severity and progression, measured by Wolfram Unified Rating Scale (WURS)<br>• Mood, measured by Kidscreen questionnaire (patients aged 8-18) and Hospital Anxiety and Depression score (HADS; adults)<br>• Quality of life, measured by Pediatric Quality of Life Inventory (PedsQL; child 8-12, parent of child 8-12, teen 13-18, parent of teen 13-18)
