# Supplementary material for: Sodium valproate, a potential repurposed treatment for the neurodegeneration in Wolfram syndrome (TREATWOLFRAM): trial protocol for a pivotal multicentre, randomised double-blind controlled trial
Source: BMJ Open. 2025 Feb 26;15(2):e091495. doi: 10.1136/bmjopen-2024-091495 (PMC11865774; doi:10.1136/bmjopen-2024-091495)
Supplement: online supplemental file 3 [file bmjopen-15-2-s003.pdf]

**Supplementary appendix 3: Example adult UK participant information sheet for the TREATWOLFRAM trial**

To be printed on hospital headed paper

# A Clinical Trial of Sodium Valproate in patients with Wolfram Syndrome

## Participant Information Sheet

Dear Participant,
We would like to invite you to take part in a non-commercial, investigator led research trial funded by the Medical Research Council (grant number: MR/P007732/1) and sponsored by the University of Birmingham, Edgbaston, Birmingham B15 2TT (the Sponsor) - TREAT WOLFRAM trial.
This information sheet is designed to help you understand what the trial is about. Please read and discuss it with anyone you wish. You may take this sheet away with you.

**Part 1** tells you the purpose of this trial
**Part 2** gives you more detailed information about the conduct of the trial.

Please ask us if there is anything that is not clear

### Part 1

### 1. What is the purpose of this Clinical Trial?

We are doing this Trial because we want to find a treatment for Wolfram syndrome.

The research team in Birmingham, UK, have studied cell models of Wolfram syndrome in the lab. They have found a medicine that protects the specialised cells and allows them to carry on working. We are, therefore testing to see if this medicine works in people with Wolfram syndrome.

We are asking if you would like to take part in a clinical trial. This means a Trial in which we invite you to randomly receive either Sodium Valproate, or a dummy medicine (placebo).

Two thirds of the participants will randomly receive Sodium Valproate, and one third, the placebo. This means that you will have a 2:1 chance of receiving Sodium Valproate rather than the placebo. Neither you nor your doctor will know which medicine you are taking. Participants are then followed up with careful measurements. Participants will be seen by the hospital/ doctor/ care team regularly to have some tests and see how you are feeling. At the end of the Trial, we will see whether people taking the Sodium Valproate have had less progression of the disease than those taking the placebo.

If Sodium Valproate works, then it will be used as a treatment for Wolfram syndrome.

You will continue to receive your usual standard of care from your doctors, whether you take part in the Trial or not.

## 2. Why have I been invited?

You have been diagnosed with Wolfram Syndrome and we believe that you may be eligible to take part in the trial.

## 3. What do I need to know about the medicine used in this Trial?

The medicine is called Sodium Valproate. Sodium Valproate is a long established medicine, licensed to treat other conditions in children and adults. It has been used worldwide for over 30 years, and there are several thousand children taking this medicine daily in the UK.

Like all medicines it has side effects. The most important safety information is that babies exposed to Sodium Valproate in their mother's womb, are at high risk of being harmed. For this reason, it is not given to women who are pregnant, or women who may become pregnant, without precautions to prevent pregnancy.

Common or very common side effects (those that affect 1 in 10 to 1 in 100 people) include nausea, tummy upset or diarrhoea (these usually disappear after a few days); headache; behaviour change; shakiness; temporary hair loss; and weight gain.

If you take part in the Trial, you will be carefully monitored for side effects, and the treatment will be stopped if necessary.

## 4. Do I have to take part?

No. Participation is voluntary. If you decide to take part, you will be asked to sign a consent form and will be given a copy to keep. You may withdraw from the trial at any time and you do not have to give any reason for your decision.

If you decide not to take part, your doctor will continue to treat you with the best means available and the standard of your care will not be affected.

If you choose to take part in the trial but later choose to withdraw, we would still like to collect information about your treatment as this will be invaluable to our research. If you have any objection to this please let your doctor know. Data already collected prior to withdrawal will be kept and analysed.

## 5. Has this drug been tested in patients before?

Sodium Valproate has been used safely in patients that need it for over 30 years. It is usually prescribed for people that have epilepsy and migraines.

**This completes Part 1 of the Information Sheet.**

**If the information in Part 1 has interested you and you are considering participation, please read the additional information in Part 2 before making any decision.**

# Part 2

## 6. What does taking part involve?

We will describe the trial and talk through this information sheet with you. You will be given time to decide whether you wish to participate in the Trial. During this time, you can discuss this with anyone you wish including your GP.

If you decide to take part you will be asked to sign a consent form and will be given a copy to keep. Following this, you will be asked to attend 11 hospital appointments and participate in 7 Telephone Appointments. The Trial will take 3 years from start to finish.

**Visit 1 - Screening visit**. You will be asked to sign a consent form to indicate you agree to take part in the trial and you will be assigned a screening number. We will then check your eligibility by asking relevant questions and performing tests, including a pregnancy test (if female). We may also ask you to give a research skin sample (optional) and a 20ml (equivalent to 4 teaspoons) blood sample for research and to use as a baseline for drug level measurements done to check treatment compliance. We may ask you to complete a questionnaire about your mood (can be done at visit 2 instead), and we will give you a diary to take home and complete to continue to record your mood, as well as a log to record any symptoms you may have until your next visit. If eligible for the imaging branch of the trial you will require a Brain scan (MRI) which will take up to 1 hour. This will provide a baseline for all follow up Brain scans (MRI) acquired as part of the trial.

__**Visit 2**__ **– Start Trial Medication**. Using the test results from visit 1 and additional tests performed during this visit (including a pregnancy test if female) your eligibility will be confirmed. You will then be officially registered into the trial, being allocated a trial number, and will receive your Trial Medication to take home and be given clear instructions on how to take it. You will also receive a Trial Medication diary where you should record the number of tablets taken every day, as well as any side effects or any missed doses.
Your treatment visit can take up to 2 hours to complete.

__**Visit 3**__ **– 3-week safety visit**. During this visit we will check that you are well and perform a blood test and pregnancy test (if female).

__**Visit 4**__ **– 6-week safety visit**. During this visit we will check that you are well and perform a blood test (including checking treatment compliance) and pregnancy test (if female). You will be asked to bring back all your Trial Medication (unused tablets, as well as empty and unused bottles) and the completed diaries. You will be given another prescription and further supply of Trial Medication to take home, and new diaries to complete.

__**Visits 5-11**__ **- follow-up visits.** These visits are designed to check your general health and do tests (including blood tests, vision tests, pregnancy tests (if female) and complete questionnaires) to determine what effect the treatment has had. For visit 6-10, you will be asked to bring back all your remaining Trial Medication received at the previous visit (including empty bottles) and the completed diaries. You will be given another prescription and further supply of Trial Medication to take home, and new diaries to complete.

**Every 12 months (at Visit 7, Visit 9 and Visit 11)** we would like to perform some additional tests and samples. This will include asking you to give a 20ml (equivalent to 4 teaspoons) research blood sample (also performed at Visit 6), a blood sample to check treatment compliance, have a brain scan (MRI), complete a sugar test with standard meal, have a bladder and hearing test if available, and a walking and balance test.

It is possible that you will be asked to attend additional unscheduled visits beyond those described if we need to re-test your blood.

The Visits 5-11 – follow up visits can take up to 2 hours to complete.

You will stop taking your trial medication at Visit 11.

To make any risks due to COVID-19 as small as possible, some of these appointments may be delivered remotely in accordance with applicable government guidelines. Trial Medication, diaries, questionnaires and pregnancy test kits (if female) may be shipped to your address. This will however be dependent on the doctors who are treating you and your hospital procedures.

**<u>Telephone Calls 1-7</u>- follow up appointments.**

We would like to telephone you in between your hospital visits to see if you are well. This will include us asking you questions about your health since the last time we spoke to you, and how you are feeling.

It is possible that you will be asked to attend additional unscheduled visits beyond those described if we feel that you need to be seen by a Doctor.

## Blood sampling procedure

A 20ml blood sample (equivalent to 4 teaspoons) will be taken at every visit. You may be asked to fast for some of the blood tests. At the screening visits they will be used to see if you are eligible for the trial, and to provide baseline values by which to compare any subsequent change after treatment. After treatment, the purpose of the blood tests are to monitor your wellbeing and health, and determine what effects the treatment has had on markers of liver function.

The method of blood samples taken during this trial are the same as that for any other normal blood sample. All blood tests may cause a momentary sharp pain on insertion of the needle and carry a risk of infection which is extremely rare.

## Research Skin sampling procedure

If you agree, we are asking you to provide us with a small skin sample at your first visit or visit 2. A small bit of skin as small as a full stop punctuation mark, will be taken by a trained health care professional. You will be given a local anaesthetic to numb the area before the sample is taken. It may be or a little uncomfortable afterwards, about the same as a blood test. The purpose is to help us with ongoing research into participants' responses to treatments. Please note that this test is optional. You can take part in the clinical trial without giving a skin sample.

## Treatment compliance blood samples

In addition to using the standard blood samples taken in this Trial, we will also take and store a blood sample to measure the level of sodium valproate in your blood. This is to meet the gold standard for clinical trials by confirming who has and who has not received the medicine under investigation. Please note that these test results will not be available to either your clinical team or yourself.

## Research blood samples

In addition to using the standard blood samples taken in this Trial, we will also take and store 20ml (equivalent to 4 teaspoons) of your blood to measure the effectiveness of treatment during the trial.

## Optional studies on left over samples

In addition to using the samples taken in this trial, we will ask your permission to store any remaining or surplus samples, and for these to be available for other medical research projects that have been approved by a Research Ethics Committee. Anyone using these samples for research will not have access to your personal details. It is difficult to predict exactly what scientific developments there may be so we cannot give precise details of what research might be done.

During and after the Trial, you are the owner of the samples. This gives you the right to have any remaining sample material destroyed (by the Sponsor) at any time. If your individual sample(s) have already been processed as part of this trial at the time of your request, the results from this analysis will remain part of the information collected as part of this clinical trial. It is important to note that there may not be any sample remaining as some of these tests use the complete sample and may result in the sample being completely destroyed as part of the test procedure. If you choose to have any remaining samples destroyed please contact your trial doctor.

Whether or not you agree to this further use will not affect your participation in the clinical trial.

| | Visit 1 (D ay -28) (-4 Week) | Visit 2 (Day 0) | TC 1 (Day 7) (Week 1) | Visit 3 (Day 21) (Week 3) | Visit 4 (Day 42) (Week 6) | Visit 5 (Day 90) (3 Month) | Visit 6 (Day 180) (6 Month) | TC 2 (Day 270) (9 Month) | Visit 7 (Day 360) (12 Month) | TC 3 (Day 450) (15 Month) | Visit 8 (Day 540) (18 Month) | TC 4 (Day 630) (21 Month) | Visit 9 (Day 720) (24 Month) | TC 5 (Day 810) (27 Month) | Visit 10 (Day 900) (30 Month) | TC 6 (Day 990) (33 Month) | Visit 11 (Day 1080) (36 Month) | TC 7 (Day 1110) (37 Month) |
|---|---|---|---|---|---|---|---|---|---|---|---|---|---|---|---|---|---|---|
| **Visit format:** | **Clinic** | **Clinic** | **TC** | **Clinic** | **Clinic** | **Clinic** | **Clinic** | **TC** | **Clinic** | **TC** | **Clinic** | **TC** | **Clinic** | **TC** | **Clinic** | **TC** | **Clinic** | **TC** |
| Informed consent | x | | | | | | | | | | | | | | | | | |
| Eligibility assessment | x | x | | | | | | | | | | | | | | | | |
| Clinical assessment | x | | | | x | | x | | x | | x | | x | | x | | x | |
| Blood tests | x | | | x | x | x | x | | x | | x | | x | | x | | x | |
| Thyroid Function Test | x | | | | | x | | | x | | | | x | | | | x | |
| Research Bloods | x | | | | | | x | | x | | | | x | | | | x | |
| Sodium valproate levels | x | | | | x | | | | x | | | | x | | | | x | |
| Pregnancy test | x | x* | | x | x | x | x | | x | | x | | x | | x | | x | |
| Skin biopsy | x | (x) | | | | | | | | | | | | | | | | |
| ECG | x | | | | | | | | | | | | | | | | | |
| Pure tone audiometry | x | (x) | | | | | | | x | | | | x | | | | x | |
| Visual Acuity: ETDRS | x | x* | | | | | x | | x | | x | | x | | x | | x | |
| Visual field Perimetry, OCT retinal thickness test, Colour Vision Test, etc | x | | | | | | | | x | | | | x | | | | x | |
| MRI scan | x | | | | | | | | x | | | | x | | | | x | |
| Mixed meal tolerance test | | x | | | | | | | x | | | | x | | | | x | |
| Urodynamics Assessment | x | | | | | | | | x | | | | x | | | | x | |

Original to be kept in the Investigator Site File, 1 copy in hospital notes, 1 copy to the patient, 1 copy to the Treat Wolfram Trial Office

| | Visit 1 (D ay -28) (-4 Week) | Visit 2 (Day 0) | TC 1 (Day 7) (Week 1) | Visit 3 (Day 21) (Week 3) | Visit 4 (Day 42) (Week 6) | Visit 5 (Day 90) (3 Month) | Visit 6 (Day 180) (6 Month) | TC 2 (Day 270) (9 Month) | Visit 7 (Day 360) (12 Month) | TC 3 (Day 450) (15 Month) | Visit 8 (Day 540) (18 Month) | TC 4 (Day 630) (21 Month) | Visit 9 (Day 720) (24 Month) | TC 5 (Day 810) (27 Month) | Visit 10 (Day 900) (30 Month) | TC 6 (Day 990) (33 Month) | Visit 11 (Day 1080) (36 Month) | TC 7 (Day 1110) (37 Month) |
|---|---|---|---|---|---|---|---|---|---|---|---|---|---|---|---|---|---|---|
| **Visit format:** | **Clinic** | **Clinic** | **TC** | **Clinic** | **Clinic** | **Clinic** | **Clinic** | **TC** | **Clinic** | **TC** | **Clinic** | **TC** | **Clinic** | **TC** | **Clinic** | **TC** | **Clinic** | **TC** |
| Wolfram Unified Rating Scale | x | | | | | | | | x | | | | x | | | | x | |
| MiniBESTest | x | (x) | | | | | | | x | | | | x | | | | x | |
| Patient Questionnaires | x | | | | | | x | | x | | x | | x | | x | | x | |
| Patient Diary collection or review | | x | x | x | x | x | x | x | x | x | x | x | x | x | x | x | x | x |
| Adverse events and concomitant medications | | x | x | x | x | x | x | x | x | x | x | x | x | x | x | x | x | x |
| Mood Questionnaire | (x) | x | | x | x | x | x | | x | | x | | x | | x | | x | |

*(x): refers to procedures that may be performed at either visit 1 or visit 2.*
** may not be repeated at visit 2 if previous test was done less than 7 days before.*

*TC:* Telephone call follow up appointment. Your Doctor or a Research Nurse will call you in between clinic visits to see how you are, to ask if you are taking any new medications and ask you about your mood diary since your last visit. The telephone call follow up appointment will take a maximum of 20 minutes to complete.

*Clinical Assessment*: This will involve you coming into hospital. An assessment of your medical history, current wellbeing, health, other medication, and any symptoms you have been experiencing. It will also include physical examination, and recording of your vital signs including pulse, blood pressure, temperature, and any others deemed necessary (including weight). Your clinical assessment should take around 30 minutes to complete.

*ECG* (electrocardiogram): This test allows the Trial doctor to see the electrical activity of your heart. Small sticky patches called electrodes are attached to your arms, legs and chest. These detect the electrical signals that make your heart beat, and transmit the signals via wires to an ECG recording machine, which in turn transcribes the signals on to paper, which a clinician (doctor or trained nurse) can interpret.

*Pregnancy test (female patients only)*: This urine test will be performed on all women of child-bearing potential who have not had a hysterectomy. You may be asked to provide a blood sample for a pregnancy serum test (Visit 1 only) and/or a urine sample at each clinic visit. For urine pregnancy tests, a nurse will test your urine sample with a dipstick and will tell you the result immediately. Alternatively, if the visit is done remotely, you will be sent pregnancy tests at home and will be given instructions to do the test yourself.

*Pure tone Audiometry*: This is a hearing test designed to help assess the type of any hearing loss that you have and will usually take around 20 minutes to complete. The Pure tone Audiometry test will involve you listening to a number of different pure tones through a pair of headphones or earplugs. When you hear the tone, you press a button or raise your hand.

*Visual acuity: ETDRS*: This is a sight test that you would receive an at opticians appointment and will take around 40 minutes to complete. If you wear glasses, you can keep them on for this test. It will involve you reading letters to the best of your ability from an ETDRS chart. An ETDRS chart typically has 5 letters per line which start off in large print and steadily decrease in size.

*Visual field Perimetry, OCT retinal thickness test, Colour Vision Test, etc…*: These are sight tests that you receive as part of your annual standard of care review. They will take around 40 minutes to complete.

*MRI Scan:* A non-invasive scan which we will use to scan your brain. This will be the same as your annual MRI scans which form part of your annual review, but we will take a few extra images for the Trial. The MRI scan will take around 40 - 60 minutes to complete. These scans will be clinically reported to your local centre by a clinical radiologist. Feedback of clinically relevant findings on the MRI will be reported back to your consultant who will then inform you of any findings. The Clinical trials unit and associate researchers from the University of Birmingham will not be made aware of the outcome of these reports unless there is an adverse finding and only after trial entry procedures have been undertaken.

*Mixed meal Tolerance test:* This is a test to see how well your pancreas can make insulin in response to food. We take a blood test for sugar and insulin levels; then give you a standard meal or milkshake to drink; then take some blood samples for sugar and insulin levels over 1 hour. The test will take around 1.5 hours to complete.

*Urodynamics assessment*: This is a standard bladder assessment patients have to assess bladder function. It will take around 20 minutes to complete.

*MiniBESTest:* This is a test to assess your balance. It will involve asking you do tasks such as walk in a straight line, and stand on one leg. It takes about 15 minutes to complete, and helps us understand how good your balance and coordination is.

*Wolfram Unified Rating Scale (WURS):* Your Doctor will perform this composite test which utilises the results of all of the above tests to see how well a person is managing with the disease.

*Patient diary:* We will issue you with a diary to take home and complete with details of when you have taken your trial medication, and how you are feeling. It is important for us to monitor your mood whilst taking the trial medication as Sodium Valproate is known to affect some people's mood. The diary will just take a minute to complete each day.

*Questionnaires*: These will involve questions about your overall quality of life and will include a Urology questionnaire, a quality of life questionnaire, a sleep questionnaire and a questionnaire about your vision. You will be asked to complete each of the paper questionnaires and you can ask for assistance if you need it. Each questionnaire will take around 15 minutes to complete.

*Mood questionnaire:* These ask specific questions about your mental wellbeing and mood. You will be asked to complete the paper questionnaire and you can ask for assistance if you need it. It is important for us to monitor your mood whilst taking the trial medication as Sodium Valproate is known to affect some people's mood. The mood questionnaire will take around 15 minutes to complete.

*Adverse events and concomitant medication:* Your Doctor will perform an assessment of your current wellbeing, health, a record of other medication you are taking, and any symptoms you have been experiencing since your last visit. This will take around 10 minutes to complete.

## 7. What are the potential benefits?

This trial is intended to see whether Sodium Valproate can slow down or halt the disease process in Wolfram syndrome.

## 8. What are the possible risks?

If Sodium Valproate is taken by women who are pregnant, there is a high risk that it will harm the unborn child. If you are female and might become pregnant, **you must** agree to use a highly effective contraceptive method prior to taking the Trial medication, and to continue this for the duration of the Trial.
You will also be asked to follow the dedicated programme called Valproate pregnancy prevention programme, regardless of the treatment you are receiving. This includes a yearly formal assessment and regular discussions with your research team study doctor whilst you are taking the Trial medication (up to 3 years).

If you are a man with a female partner of childbearing potential, you must agree to the use of condoms (unless you have had a vasectomy) and to the use of a highly effective contraception by your female partner prior to taking the Trial medication, and you must both continue this for the duration of the Trial.

Highly effective contraceptive methods include any of the following methods:

- Combined (estrogen and progestogen containing) hormonal contraception (Oral, Intravaginal or Transdermal)
- Progestogen-only hormonal contraception (Oral, Injectable or implantable):
- Intrauterine device (IUD)
- Intrauterine hormone-releasing system (IUS)
- Bilateral tubal occlusion (female sterilisation)
- Sexual abstinence

If you or your partner become(s) pregnant while you are taking the Sodium Valproate, you must tell your doctor immediately.

Severe liver damage including liver failure, sometimes resulting in fatalities, has been very rarely reported. Early symptoms may include sudden onset of tiredness, lack of energy, and drowsiness. If you develop any of these, you should let your doctor know immediately.

If you have a concern about any part of this Trial, please speak to your doctor or one of the Trial doctors. We will do our best to answer these questions (see contact details on first page). If you still

have concerns, you can raise these using the NHS complaints procedure. You can ask how to do this at your hospital.

If something goes wrong while you are taking part in this Trial, we will do everything we can to look after you. We will make sure you are closely supervised while taking part in the Trial.

You are free to stop at any time without a reason.

If you decide to stop taking part in the Trial, you will only need to inform your doctor and he or she will withdraw you from the Trial. This will not affect your standard of care.

## 9. Prohibited medication

Whilst taking part in the trial you will be asked to let your doctors know if you are taking any other medicines.

## 10. What happens when the trial stops?

You will return to clinical care as normal. Your clinical team will continue to keep you informed about all treatments (standard of care or experimental) from clinic.

## 11. Expenses and payments

When you come into hospital for your trial visits, reasonable travel expenses can be reimbursed, up to a maximum of £30 per treatment visit on production of receipts.  Travel by car will be reimbursed at the standard NHS patient reimbursement rate of your local NHS trust (typically 23 pence per mile).

If you feel that it is likely that your expenses are going to be higher than the £30.00 per treatment visit, due to the distance that you have to travel to the hospital, car parking charges or any other reason, then please discuss this with the local hospital team. Depending on individual circumstances, it may be possible to find additional ways to help with extra travel or accommodation costs.

Depending on local hospital arrangements, food and drinks will be provided by the hospital during the treatment visits. Please ask your local research team for details.

## 12. What if relevant new information becomes available?

Sometimes we get new information about the treatment being studied. If this happens, your Trial doctor will inform you and discuss with you whether you should continue in the trial. If you decide not to carry on, your trial doctor will make arrangements for your normal care to continue. If you decide to continue in the trial, he/she may ask you to sign an updated consent form. It is possible that your trial doctor might suggest you withdraw from the trial. He/she will explain the reasons and arrange for your care to continue. If the trial is stopped for any other reason, we will tell you and arrange your continuing care.

## 13. What will happen if I don't want to carry on with the Trial?

If you do not wish to carry on with the trial, you will have the option to withdraw entirely from the trial or you may wish to withdraw from treatment only and carry on being assessed within the trial.

If you choose to take part in the trial but later withdraw, we would still like to collect information about your treatment as this will be valuable to our research. If you have any objection to this please let your doctor know when you decide to withdraw from the trial. Data already collected prior to withdrawal will be kept and analysed.

## 14. What if there is a problem?

If you have a concern about any aspect of this trial, you should ask to speak with the trial doctor who will do their best to answer your questions (see contact number at end of form). If you remain unhappy and wish to complain formally, you can do this through your hospital's Patient Advice and Liaison Services (PALS); they can be contacted by:

(Insert local contact details).

In the event that something does go wrong and you are harmed during the trial there are no special compensation arrangements. If you are harmed and this is due to someone's negligence then you may have grounds for legal action for compensation against the Sponsor of the trial (University of Birmingham) or the NHS Trust but you may have to pay your legal costs. NHS Trust and Non-Trust Hospitals have a duty of care to patients treated, whether or not the patient is taking part in a clinical trial and the normal NHS complaints mechanisms will still be available to you (if appropriate).

## 15. What will happen to the results of the Clinical Trial?

At the end of the Trial, the information collected will be analysed and published in recognised medical journals. The identity of the patients who took part in the trial will remain confidential.

Should you wish to discuss the results of the trial, you should contact your trial Doctor; you will have the opportunity if you wish to be informed of the results of the trial once fully analysed.

## 16. Who has reviewed this Clinical Trial?

All research in the NHS is looked at by independent group of people called a Research Ethics Committee to protect your safety, rights, wellbeing and dignity. This Trial has been reviewed and given favourable opinion by the West Of Scotland Research Ethics Committee 1 and by the NHS Health Research Authority. While the Trial is ongoing the results will be reviewed by an independent Data Monitoring Committee (DMC) to ensure that it is appropriate to continue with the Trial.

## 17. Will my taking part be kept confidential and secure?

Yes. All information collected about you for this trial will be subject to the General Data Protection Regulation (GDPR) and Data Protection Act 2018 for health and social care research and will be kept

strictly confidential. University of Birmingham is the sponsor for this study. The University of Birmingham will be using information from you and/or your medical records in order to undertake this study and will act as the data controller for this study. This means that the University of Birmingham are responsible for looking after your information and using it properly.
In order to carry out the research project described above, we will need to collect information about you, and some of this information will be your personal data. Under data protection law, we have to provide you with very specific information about what we do with your data and about your rights. We have set out below the key information you need to know about how we will use your personal data.

More information on how the University processes personal data can be found on the University's website on the page called 'Data Protection - How the University Uses Your Data' (https://www.birmingham.ac.uk/privacy/index.aspx).

Additional information about the CRCTU (Clinical Trials Unit) can be found here:
https://www.birmingham.ac.uk/research/activity/mds/trials/crctu/index.aspx

**Who is the Data Controller?**
The University of Birmingham, Edgbaston, Birmingham B15 2TT is the data controller for the personal data that we process in relation to you.

**What data are we processing and for what purpose will we use it?**
We will collect and process your personal data to conduct the research project, as explained in the Participant Information Sheet.

**What is our legal basis for processing your data?**
The legal justification we have under data protection law for processing your personal data is that it is necessary for our research, which is a task we carry out in the public interest.

**Who will my personal data be shared with?**
For the purposes of the research project, we will need to share your personal data with external laboratories so that they can correctly identify the samples and report the results. We have appropriate agreements in place with them to protect and safeguard your data and will only use minimum personally-identifiable information possible.
Sometimes, external organisations assist us with processing your information, for example, in providing IT support. These organisations act on our behalf in accordance with our instructions and do not process your data for any purpose over and above what we have asked them to do. We make sure we have appropriate contracts in place with them to protect and safeguard your data. If your personal data are transferred outside the European Union (for example, if one of our partners is based outside the EU or we use a cloud-based app with servers based outside the EU), we make sure that appropriate safeguards are in place to ensure the confidentiality and security of your personal data.

The NHS will use your name and contact details to contact you about the research study, and make sure that relevant information about the study is recorded for your care, and to oversee the quality of the trial. With your permission your research doctor will notify your GP that you intend to participate in the trial. They will also send a copy of your signed consent form in the post to the Trials Office. Your individual contact details (Home address, Name and potentially telephone number) will remain within the NHS and will not be disclosed to any third party except the organisation responsible for the delivery of the medication and study supplies to your home address in case of remote appointments. No additional information will be supplied to third parties outside the specific requirements to provide this service.

In the Trials Office you will be identified by a unique trial number. In routine communication between your hospital and the Trials Office you will only be identified by trial number, initials and date of birth. This is known as 'linked anonymised data' where your data is anonymous to the people who receive and hold it (the Trials office) but it contains information that will allow the suppliers of the data (your Hospital) to identify people from it. Data may be provided to the Trials Office on paper or electronically.

Due to the rarity of a condition, like Wolfram Syndrome, it is sometimes possible to deduce an individual's identity through combinations of information. All individuals who have access to your information have a duty of confidentiality to you and under no circumstances will you be identified in any way in any report, presentation or publication arising from this trial. All necessary steps will be taken to ensure that your identity cannot be deduced from any combination of data items.

By taking part in the trial you will be agreeing to allow research staff from the Trials Office to look at the trial records, including your medical records. It may be necessary to allow authorised personnel from government regulatory agencies (e.g. Medicines and Healthcare products Regulatory Agency (MHRA), the Sponsor and/or NHS bodies to have access to information about you. This is to ensure that the trial is being conducted to the highest possible standards.

In addition, if you have provided blood or skin samples for the trial, your screening or trial number and month/year of birth may be passed on to personnel at external (to your local hospital) blood testing laboratories to help them identify the samples.

Throughout the course of your treatment, blood samples will be taken for research and analysis. Samples that will be sent for analysis include tests to see if the treatment is working, and to grow cells to study how the medicine is working. These will then be labelled and transported by courier to laboratories. Research blood samples will be sent to the NIHR Wellcome Trust Clinical Research Facility, Birmingham, and the University of Birmingham. Samples for analysis may be sent to different laboratories in the United Kingdom and elsewhere (including University of Birmingham). The only

identifiable information on the labels will be the screening or trial number and month/year of birth. These details will remain confidential within the trial team.

If you give your consent, any samples leftover at the end of analysis may be kept for future ethically approved research.

Data collected during the Trial (i.e. results of research blood samples) may be transferred, for the purpose of processing and analysing, to associated researchers and independent specialist reviewers within the European Economic Area and the United States of America. These details will remain confidential within the trial team.

Brain Scans (MRI) will be sent to the University of Birmingham for research analysis. All identifiable information will be removed before transfer from the acquiring site to the associated research teams. Your Brain scans will only be identified via your Trial Number (or screening number) and the date of your scan.

Additionally, when you agree to take part in a research study, the information about your health and care may be provided to researchers running other research studies in the University of Birmingham and in other organisations. These organisations may be universities, NHS organisations or companies involved in health and care research in this country or abroad. Your information will only be used by organisations and researchers to conduct research in accordance with the UK Policy Framework for Health and Social Care Research.
Your information could be used for research in any aspect of health or care, and could be combined with information about you from other sources held by researchers, the NHS or government.
Where this information could identify you, the information will be held securely with strict arrangements about who can access the information. The information will only be used for the purpose of health and care research, or to contact you about future opportunities to participate in research. It will not be used to make decisions about future services available to you, such as insurance.
Where there is a risk that you can be identified your data will only be used in research that has been independently reviewed by an ethics committee.

**How will my personal data be kept secure?**

The University takes great care to ensure that personal data is handled, stored and disposed of confidentially and securely. Our staff receive regular data protection training, and the University has put in place organisational and technical measures so that personal data is processed in accordance with the data protection principles set out in data protection law.

The University has an Information Security Management System based on ISO27001 with a range of controls covering the protection of personal information. Annual security awareness training is mandatory for staff and the University is accredited under the NHS Information Governance Toolkit,

the Payment Card Industry Data Security Standard and is in the process of gaining Cyber Essentials Plus for defined services.

In relation to this project, all information collected by the Sponsor will be securely stored at the Cancer Research UK Clinical Trials Unit at the University of Birmingham (the Trials Office) on paper and electronically and will only be accessible by authorised personnel. The only people in the University of Birmingham who will have access to information that identifies you will be people who manage the study or audit the data collection process.

**How long will my personal data be kept?**

The University of Birmingham and the NHS will keep identifiable information about you for at least 25 years after the study has finished, to allow the results of the study to be verified if needed.

If you withdraw from the project, we will keep the information we have already obtained but, to safeguard your rights, we will use the minimum personally-identifiable information possible.

**Your rights in relation to your data**

Your rights to access, change, or move your information are limited, as we need to manage your information in specific ways in order for the research to be reliable and accurate. If you withdraw from the study, we will keep the information about you that we have already obtained but, to safeguard your rights, we will use the minimum personally-identifiable information possible.

If you would like more information on your rights, would like to exercise any right or have any queries relating to our processing of your personal data, please contact:

> The Information Compliance Manager, Legal Services, The University of Birmingham, Edgbaston, Birmingham B15 2TT
>
> Email: dataprotection@contacts.bham.ac.uk Telephone: +44 (0)121 414 3916

If you wish to make a complaint about how your data is being or has been processed, please contact our Data Protection Officer.

> The Data Protection Officer, Legal Services, The University of Birmingham, Edgbaston, Birmingham B15 2TT
>
> Email: dataprotection@contacts.bham.ac.uk Telephone: +44 (0)121 414 3916

You also have a right to complain to the Information Commissioner's Office (ICO) about the way in which we process your personal data. You can make a complaint using the ICO's website.

## 18. Further information and contact details

If you have any questions or concerns about your disease or this clinical trial, please discuss them with your doctor. You can get in touch with the doctors and nurses at any time to discuss any doubts or worries you may have about the trial, and we will give you a card with contact details. Contact details are also shown below:

| | | |
|---|---|---|
| Trial Doctor | Dr (insert name) | Tel: (insert number) |
| Research Nurse(s) | (insert name) | Tel: (insert number) |
| Hospital Details | (insert name and address of hospital) | |
| Registrar out of hours | Available via hospital switchboard | |
