# Supplementary material for: Sodium valproate, a potential repurposed treatment for the neurodegeneration in Wolfram syndrome (TREATWOLFRAM): trial protocol for a pivotal multicentre, randomised double-blind controlled trial
Source: BMJ Open. 2025 Feb 26;15(2):e091495. doi: 10.1136/bmjopen-2024-091495 (PMC11865774; doi:10.1136/bmjopen-2024-091495)
Supplement: online supplemental file 4 [file bmjopen-15-2-s004.pdf]

Supplementary appendix 4: Example adult informed consent form for the TREATWOLFRAM trial

To be printed on hospital headed paper

A Clinical Trial of Sodium Valproate in patients with Wolfram Syndrome

**Site:**	....	**Patient Trial Number:**	☐☐☐
**Principal Investigator:**	....	**Trial Reference Number:**	2017-001215-37

Please initial each box

1. I confirm that I have read and understand the **Patient Information Sheet for Adults** (version .... dated.........................) for the above trial. I have had the opportunity to consider the information, ask questions and have had these answered satisfactorily. ☐
2. I understand that my participation is voluntary and that I am free to withdraw at any time without giving any reason, without my medical care or legal rights being affected. ☐
3. I give permission for my name, date of birth, gender and NHS number to be given to the Trials Office when I am randomised to the trial as well as a copy of this consent form. ☐
4. I understand that relevant sections of my medical notes and data collected during the trial may be looked at by individuals from the Trials Office, regulatory authorities, Sponsors and/or NHS bodies, where it is relevant to my taking part in this research. I give permission for these individuals to have access to my records. ☐
5. I understand that samples of my blood will be collected, stored and undergo analysis for the purposes of this trial and to understand Wolfram Syndrome. ☐
6. I understand that anonymised data and samples from the trial may be provided to other 3rd parties (e.g. pharmaceutical companies or other academic institutions) for research, safety monitoring or licensing purposes. This data will be provided to countries inside and outside the European Economic Area. My identity will remain anonymous. ☐
7. I understand that my MRI scans will be transferred to associate researchers at the University of Birmingham for Research analysis ☐
8. I understand that my local centre will be carrying out clinical reporting of my MRI scans and the findings from these reports will be communicated to me by my normal clinical team and not via the Trials Office. ☐

CRCTU-ICF-QCD-001, version 2.0

Original to be kept in the Investigator Site File, 1 copy in hospital notes, 1 copy to the patient, 1 copy to the Trials Office

9. I agree to my GP being informed of my participation in this trial and to my GP being sent a copy of this consent form.

10. I agree to take part in the above trial.

The following are optional to <u>UK patients only</u> and will not affect entry into the trial, please initial in each box:

	No	Yes
OPTIONAL – I agree to my health records being flagged through the NHS Information Centre for Health and Social Care service to assist with long term follow up data collection.		
OPTIONAL – I consent for any remaining research blood samples being stored and used for future research purposes including stem cell research, genetic studies, research which may use in vitro models, and research involving private or commercial companies.		
OPTIONAL – I agree to the collection, storage and analysis of skin samples to understand Wolfram syndrome. I also consent for any remaining skin samples being stored and used for future research purposes including stem cell research, genetic studies, research which may use in vitro models, and research involving private or commercial companies.		

**Name of patient**	**Date**	**Signature**
**Name of person taking consent**<br>You must have signed the<br>Site Signature & Delegation Log	**Date**	**Signature**

CRCTU-ICF-QCD-001, version 2.0

Original to be kept in the Investigator Site File, 1 copy in hospital notes, 1 copy to the patient, 1 copy to the Trials Office
