# Supplementary material for: Sodium valproate, a potential repurposed treatment for the neurodegeneration in Wolfram syndrome (TREATWOLFRAM): trial protocol for a pivotal multicentre, randomised double-blind controlled trial
Source: BMJ Open. 2025 Feb 26;15(2):e091495. doi: 10.1136/bmjopen-2024-091495 (PMC11865774; doi:10.1136/bmjopen-2024-091495)
Supplement: online supplemental file 5 [file bmjopen-15-2-s005.pdf]

Supplementary appendix 5. TREATWOLFRAM schedule of events

				Visits 3 and 4 +/- 7 days		All Telephone Calls and Visits from Visit 5 Onwards: +/- 14 days												
	**Visit 1** (D ay -28) (-4 Week)	**Visit 2** (Day 0)	**TC 1** (Day 7) (Week 1)	**Visit 3** (Day 21) (Week 3)	**Visit 4** (Day 42) (6 Week)	**Visit 5** (Day 90) (Week 12/ 3 Month)	**Visit 6** (Day 180) (6 Month)	**TC 2** (Day 270) (9 Month)	**Visit 7** (Day 360) (12 Month)	**TC 3** (Day 450) (15 Month)	**Visit 8** (Day 540) (18 Month)	**TC 4** (Day 630) (21 Month)	**Visit 9** (Day 720) (24 Month)	**TC 5** (Day 810) (27 Month)	**Visit 10** (Day 900) (30 Month)	**TC 6** (Day 990) (33 Month)	**Visit 11** (Day 1080) (36 Month)	**TC 7** (Day 1110) (37 Month)
**Visit format:**	**Clinic**	**Clinic**	**TC**	**Clinic[#COV]**	**Clinic**	**Clinic[#COV]**	**Clinic**	**TC**	**Clinic**	**TC**	**Clinic**	**TC**	**Clinic**	**TC**	**Clinic**	**TC**	**Clinic**	**TC**
Informed consent	X																	
Demography	X																	
Medical history	X																	
Inclusion/ exclusion criteria	X	X																
Physical examination [1] [COV]	X				X		X		X		X		X		X		X	
Biochemistry [2]	X			X	X	X	X		X		X		X		X		X	
Haematology [3]	X			X	X	X	X		X		X		X		X		X	
Thyroid function tests [4]	X					X			X				X				X	
Research bloods [5]	X						X		X				X				X	
Sodium valproate levels [6]	X				X[COV]				X				X				X	
Pregnancy test [7] [COV]	X	(X)		X	X	X	X		X		X		X		X		X	
Skin biopsy [8] [‡]	X	(X)																
ECG [9]	X																	
Pure tone audiometry [10†]	X[COV]	(X[COV])							X				X				X	
Visual acuity: ETDRS[11]	X	(X)					X		X		X		X		X		X	
Visual field Perimetry, OCT retinal thickness	X								X				X				X	

				Visits 3 and 4 +/- 7 days		All Telephone Calls and Visits from Visit 5 Onwards: +/- 14 days												
	**Visit 1** (D ay -28) (-4 Week)	**Visit 2** (Day 0)	**TC 1** (Day 7) (Week 1)	**Visit 3** (Day 21) (Week 3)	**Visit 4** (Day 42) (6 Week)	**Visit 5** (Day 90) (Week 12/ 3 Month)	**Visit 6** (Day 180) (6 Month)	**TC 2** (Day 270) (9 Month)	**Visit 7** (Day 360) (12 Month)	**TC 3** (Day 450) (15 Month)	**Visit 8** (Day 540) (18 Month)	**TC 4** (Day 630) (21 Month)	**Visit 9** (Day 720) (24 Month)	**TC 5** (Day 810) (27 Month)	**Visit 10** (Day 900) (30 Month)	**TC 6** (Day 990) (33 Month)	**Visit 11** (Day 1080) (36 Month)	**TC 7** (Day 1110) (37 Month)
**Visit format:**	**Clinic**	**Clinic**	**TC**	**Clinic**[#COV]	**Clinic**	**Clinic**[#COV]	**Clinic**	**TC**	**Clinic**	**TC**	**Clinic**	**TC**	**Clinic**	**TC**	**Clinic**	**TC**	**Clinic**	**TC**
test, colour vision test, etc [12†‡]																		
MRI scan [13]	X*								X				X				X	
Mixed meal tolerance test (MMTT), or equivalent[14]		X							X				X				X	
Urodynamics assessment [†‡]	X								X				X				X	
Wolfram Unified Rating Scale (WURS)[‡]	X								X				X				X	
Mini-BESTest [15‡]	X[COV]	(X[COV])							X				X				X	
Patient questionnaires [16 COV **]	X						X		X		X		X		X		X	
Patient diaries collection and/or review [17]	Issue symptoms log and mood diary	X	X	X	X	X	X	X	X	X	X	X	X	X	X	X	X	X
Prescription [18]		X			X		X		X		X		X		X			
Adverse events and concomitant medications [19]		X	X	X	X	X	X	X	X	X	X	X	X	X	X	X	X	X
Mood questionnaire [20 COV **]	(X)	X		X	X	X	X		X		X		X		X		X	
End of treatment [21]																	X	
End of trial [22]																		X

ECG, electrocardiogram; ETDRS, early treatment diabetic retinopathy study; OCT, optical coherence tomography; Mini-BESTest, mini balance evaluation systems test; MRI, magnetic resonance imaging; TC, telephone call.

Key:

[1] Physical examination will include: general examination, cardiovascular including blood pressure; respiratory; abdominal; neurological examination; Vital signs, and Tanner Pubertal stage at visit 1 and 11 only if patient has not yet completed puberty (additional staging may be performed as per local practice and only if deemed clinically appropriate by Investigator). In the event of a clinic visit being performed remotely because of COVID-19 restrictions at site, Physical examination is not required as it cannot be performed.

[2] Biochemistry tests to include: blood (serum or plasma) urea, creatinine, electrolytes (sodium, potassium), bone chemistry (plasma calcium, phosphate, alkaline phosphatase , vitamin D, parathyroid hormone), lactate, random glucose, random plasma osmolarity and urine osmolarity, glycated haemoglobin, liver function tests (total bilirubin, alanine transaminase, aspartate transaminase, gamma glutamyl transferase), coagulation (prothrombin time, thrombin time, activated partial thromboplastin time, international normalised ratio, fibrinogen), ammonia, amylase.

[3] Haematology tests to include haemoglobin, white count and differential, platelet count.

[4] Thyroid function tests (TFT) to include thyroid-stimulating hormone (TSH), free triiodothyronine (FT3) and free thyroxine (FT4). In the UK, FT3 is only mandated if the other 2 tests are abnormal. TFT may also be performed in case of early IMP discontinuation if clinically indicated (see footnote 21).

[5] Research blood sampling at UK sites only: peripheral blood monocyte cell (PBMC) biomarker assay ($p21^{cip1}$ and upstream regulators), blood samples for expression arrays and single nucleotide polymorphism (SNP) array, and research saved samples for future studies. If there are more than 28 days between Research blood samples collection and randomisation, samples do not need to be repeated after re-consent.

[6] Sodium valproate levels measured in blood samples for treatment compliance checks – **mandatory for all patients**. If a sample can't be collected at Visit 4 due to COVID-19 restrictions at site, a sample should be collected at the next available opportunity. A sample should also be collected: in case of an Early IMP Discontinuation visit performed on site (see footnote 21); after 6 weeks when restarting IMP restart after a treatment discontinuation.

[7] Pregnancy tests to be performed at each clinic visit on female patients of childbearing potential. For visit 1, a serum (preferred) or highly sensitive urine pregnancy test should be performed. Urine pregnancy tests are acceptable for all subsequent visits. Visit 2 pregnancy test is optional if previous test performed less than 7 days ago. Additional pregnancy tests may be performed according to local Valproate pregnancy prevention program. If a clinic visit is done remotely due to COVID-19 restrictions at site, self-certified home pregnancy test is allowed if permitted by local Competent Authority. In case of early IMP discontinuation, Pregnancy testing should be carried out for female patients as per the schedule of events during the 2 months following the last dose of IMP. Pregnancy testing is not required after this while patient remains off treatment.

[8] Skin biopsy sample for fibroblast culture – UK patients only, subject to consent. Skin biopsy can be performed at **visit 1 <u>or</u> visit 2.** If there are more than 28 days between Skin biopsy and randomisation, biopsy does not need to be repeated after re-consent.

[9] 12 lead ECG. To be performed at Visit 1, and subsequently only if deemed clinically appropriate by Investigator.

[10] Initial auditory assessment including Pure Tone Audiometry can be performed at **visit 1 <u>or</u> visit 2** prior to start of treatment during COVID-19 pandemic.

[11] Visual acuity should be repeated at Visit 2 if Visit 1 assessment was performed more than 7 days ago **or** if the LogMAR score at Visit 1 for the better eye was between 1.45 and 1.6 on an ETDRS chart, with or without corrected vision. If the latter, the visit 1 LogMAR score central review should be expedited. Assessment optional at Visit 2 otherwise.

[12] Visual field can be assessed by the local centre standard process, but the same technique must be used throughout the patient participation to the trial.

[13] MRI scan measuring brain volumes. Patients who do not pass local safety screening for MR scanning on a whole body 3T MR scanner or who have metallic implants in the vicinity of the scanned area (brain) should not perform this assessment.

*For visit 1, an MRI conducted as part of standard care may be used only if it is in accordance with the TREATWOLFRAM Imaging manual and if it was performed within 180 days prior to visit 1 and before the start of trial treatment (visit 2).

[14] Mixed meal tolerance test (MMTT), or equivalent is for UK patients only. If for any reason it is not possible to complete a MMTT, then paired fasting or random glucose and C-peptide may be collected.

[15] Mini-BESTest initial assessment can be performed at **visit 1 or visit 2** during COVID-19 pandemic.

[16] Patient questionnaires, to include: International Consultation on Incontinence Questionnaires (ICIQ): ICIQ-FLUTS (adult females), ICIQ-MLUTS (adult males), ICIQ-CLUTS (children, caregiver form); Quality of Life questionnaires: PedsQL (child 8-12, parent of child 8-12, teen 13-18, parent of teen 13-18), Sleep Questionnaires (Sleep-related Breathing disorder scale extracted from the Pediatric Sleep Questionnaire - referred to as PSQ - parent report for those under 18; and Pittsburgh Sleep Quality Index (PSQI) Self-Report for adults) and Visual function questionnaires (VQoL-C (children 8-12), VQoL-YP (young people 13-18), FVQ-C (children 8-12), FVQ-YP (young people 13-18) and VFQ25 self report or interviewer-administered report for adults). During COVID-19

pandemic, it is permitted to have questionnaires completed at home by the patient/parent/carer in order to minimise time spent in hospital during the visit.

[17] Patient diaries – to include trial medication diary, symptoms log, mood diary. At visit 1, issue symptoms log and mood diary to the patient. At every prescription visit, issue trial medication diary/-ies to the patient (initial 6-week diary at visit 2, monthly diaries thereafter).

[18] Prescription - The first prescription issued at visit 2 will be for a 6-week supply of the Investigational Medicinal Product (IMP). The prescription at visit 4 will be for a 4½-month supply of the IMP. All prescriptions thereafter will be for a 6-month supply of the IMP to reduce the burden of additional hospital visits to the patient. All prescriptions should also take into account the protocol-defined visit windows to cover IMP requirements until the next prescription visit.

[19] Adverse event and concomitant medications recording.

[20] Mood questionnaires: Kidscreen (Patients aged 8-18) or Hospital Anxiety and Depression Score (HADS - adults). Mood questionnaires can be administered either at **visit 1 or visit 2**, provided eligibility has been reviewed. During COVID-19 pandemic, it is permitted to have Kidscreen mood questionnaires completed at home by the patient in order to minimise time spent in hospital during the visit or in case of remote visit. HADS questionnaire must be administered at hospital site.

[21] End of treatment visit. If a patient discontinues treatment early for more than 7 continuous days either due to an adverse event or patient's decision before Visit 11, an (unscheduled) face-to-face safety visit should be arranged within 4 weeks of the site being informed (unless patient unable to attend for medical reasons – consider remote visit or request for additional medical information from GP/local hospital). This **Early IMP Discontinuation Visit** should

include: a physical examination, biochemistry and haematology tests, thyroid function tests (if clinically indicated), a pregnancy test for female patients of childbearing potential, collection of blood samples for measurement of sodium valproate levels, ECG (if clinically indicated), collected and review of all patient diaries, recording of Adverse events and Concomitant Medications, administration of Mood questionnaire (see footnote 20).

[22] If a patient discontinues treatment early for more than 7 continuous days, a follow up telephone call should occur within 28 days of the Early IMP Discontinuation Visit described in footnote 21 +/- 14 days.

[#] Unscheduled visit or unscheduled blood test, and non-prescription visits (Visits 3 and 5) can be performed at a Shared Care centre or equivalent.

[†] Service-based tests to be done if facilities permit but not essential.

[‡] Optional for non-UK sites.

** For non-UK sites, questionnaires to be performed only if there is a validated translated version available in the country.

[COV] Assessment or visit whose requirement/delivery is altered in case of COVID-19 restrictions at site (please also refer to each specific assessment footnote for details). Visits 3 and 5 may be delivered remotely and safety blood samples collected at an off-site premise if the patient is unable to come into the Hospital. The investigator should ideally obtain and review the results within 3 working days and no later than 5 working days. In case of abnormal results, a clinic visit will need to be scheduled at the primary treatment centre within 14 days, or earlier depending on the seriousness of the abnormal results.
