# Supplementary material for: Sodium valproate, a potential repurposed treatment for the neurodegeneration in Wolfram syndrome (TREATWOLFRAM): trial protocol for a pivotal multicentre, randomised double-blind controlled trial
Source: BMJ Open. 2025 Feb 26;15(2):e091495. doi: 10.1136/bmjopen-2024-091495 (PMC11865774; doi:10.1136/bmjopen-2024-091495)
Supplement: online supplemental file 6 [file bmjopen-15-2-s006.pdf]

Supplementary appendix 6: Prohibited concomitant mediations and those to be used with caution within the TREATWOLFRAM trial

Drug name	Group	Interaction with Sodium Valproate	Contra-indication	Instructions
**Prohibited**				
Aspirin	Salicylates / salicylic acid	Employ the same metabolic pathway as Sodium Valproate – should not be used concurrently – increased risk of liver Dysfunction/damage – has resulted in fatalities<br><br>Possible increase in free valproic Acid plasma levels	Predominantly under-16 years of age	Consider other analgesics. Careful monitoring of liver function (liver functions tests) and clinical signs i.e. presence of Jaundice.
**To be used with caution**				
Imipenem – cilastitin<br>Meropenem<br>Doripenem<br>Ertapenem<br>Panipenem	Carbepenem agents – antibiotic agents	Not recommended – decreases blood levels of Valproic acid (60%-100%)	Not recommended	Consider use of alternative antibiotic classes – if no alternative additional clinical monitoring required in line with institute standard practice.
Olanzapine<br>Lithium	Antipsychotics, MAO inhibitors, Anti-depressants and Benzodiazepines	May increase the risk of certain adverse events<br>Valproic acid may decrease Olanzapine plasma concentration	Use with caution	Careful clinical monitoring of adverse events
Phenobarbital	Anticonvulsant/ hypnotic drug	Sodium valproate increases plasma concentrations (due to inhibition of hepatic catabolism) – *Sedation may occur, particularly in children*<br><br>Valproic acid metabolite levels may be increased in the case of concomitant use.	Use with caution	Careful clinical monitoring of the first 15 days of concomitant use and IMMEDIATE reduction of Phenobarbital if sedation occurs. Monitor for signs and symptoms of hyperammonaemia
Primidone	Anticonvulsant	Sodium Valproate increases Primidone form- leading to	Use with caution	Careful clinical monitoring of adverse events -

Drug name	Group	Interaction with Sodium Valproate	Contra-indication	Instructions
		possible overdose symptoms / increase in adverse effects (i.e. sedation)		consider dosage modification when appropriate
Phenytoin	Anticonvulsant	Sodium Valproate increases Phenytoin form- leading to possible overdose symptoms	Use with caution	Careful clinical monitoring of adverse events including signs and symptoms of hyperammonaemia
Carbamazepine	Anticonvulsant	Potential for increased toxic effects/adverse events	Use with caution	Careful clinical monitoring of adverse events – consider dosage modification when appropriate
Lamotrigine	Anticonvulsant	Sodium Valproate reduces the metabolism of Lamotrigine – can lead to increased toxicity	Use with caution	Careful clinical monitoring of adverse events– consider dosage modification when appropriate
Rufinamide	Anticonvulsant	Valproate increases the exposure to rufinamide – Caution should be exercised particularly in children.	Use with caution	Careful clinical monitoring of adverse events- consider dosage modification when appropriate
Propofol	Anaesthetic drug	Valproate potentially increases the concentration of propofol.	Use with caution	Careful clinical monitoring of adverse events- consider reducing propofol when appropriate
Zidovudine	Antiretroviral	Sodium Valproate may raise Zidovudine plasma concentration leading to increased Zidovudine toxicity	Use with caution	Careful clinical monitoring of adverse events- consider dosage modification (decrease) when appropriate.
Nimodipine	Calcium channel blocker	Concomitant treatment can increase exposure to nimodipine by 50%. Nimodipine should be decreased in case of hypotension.	Use with caution	Careful clinical monitoring of adverse events- consider dosage modification (decrease) when appropriate
➢ Warfarin<br>➢ Other Coumarin anticoagulants	Vitamin-K dependent factor anticoagulants	Potential to increase anticoagulant effect.	Use with caution	Careful clinical monitoring of prothrombin time
Felbamate	Anticonvulsant	Potential to decrease Sodium Valproate	Use with caution	*Patients on the Treat Wolfram trial will be on active or*

Drug name	Group	Interaction with Sodium Valproate	Contra-indication	Instructions
		clearance by 22% to 50%<br>Valproic acid may decrease the felbamate mean clearance by up to 16%.		*placebo treatment – so no clinical guidance is relevant outside protocol defined procedures.*
Cimetidine	Histamine Blockers	Valproate acid plasma levels may be increased	Use with Caution	*Patients on the Treat Wolfram trial will be on active or placebo treatment – so no clinical guidance is relevant outside protocol defined procedures.* Potential for increased adverse events
➢ Mefloquine<br>➢ Chloroquine	Malaria medication	Potential for increased metabolism of sodium valproate	Use with caution	*Patients on the Treat Wolfram trial will be on active or placebo treatment – so no clinical guidance is relevant outside protocol defined procedures.*
Erythromycin	Antibiotic	Valproate acid plasma levels may be increased	Use with Caution	*Patients on the Treat Wolfram trial will be on active or placebo treatment – so no clinical guidance is relevant outside protocol defined procedures.* Potential for increased adverse events
➢ Lopinavir Ritonavir	Protease inhibitors	Decrease valproate plasma level	Use with caution	*Patients on the TREATWOLFRAM trial will be on active or placebo treatment – so no clinical guidance is relevant outside protocol defined procedures.*
1. Quetiapine	Anti-psychotic	Increase risk of neutropenia/ leucopenia	Use with caution	Careful clinical monitoring of adverse events
2. Cholestyramine	Bile Acid Sequestrant	Potential for decreased plasma level of valproate	Use with caution	*Patients on the TREATWOLFRAM trial will be on active or placebo treatment – so no*

**Drug name**	**Group**	**Interaction with Sodium Valproate**	**Contra-indication**	**Instructions**
				clinical guidance is relevant outside protocol defined procedures.
3. Rifampicin	Antibiotic	Valproate acid plasma levels maybe decreased	Use with caution	Consider use of alternative antibiotic classes – if no alternative additional clinical monitoring required in line with institute standard practice.
➢ Topiramate<br>4. Acetazolamide	Anticonvulsant drug	Combined use of with Sodium valproate has been associated with Encephalopathy and/or Hyperammonaemia	Use with caution	Careful clinical monitoring of adverse events (signs and symptoms)
1. Oestrogen containing products (including oestrogen-containing hormonal contraceptives)		May increase clearance of valproate	Use with caution	*Patients on the TREATWOLFRAM trial will be on active or placebo treatment – so no clinical guidance is relevant outside protocol defined procedures.*
