# Supplementary figures and images for: Sodium valproate, a potential repurposed treatment for the neurodegeneration in Wolfram syndrome (TREATWOLFRAM): trial protocol for a pivotal multicentre, randomised double-blind controlled trial
Source: BMJ Open. 2025 Feb 26;15(2):e091495. doi: 10.1136/bmjopen-2024-091495 (PMC11865774; doi:10.1136/bmjopen-2024-091495)

## Supplementary appendix 8: Cumulative recruitment during the TREATWOLFRAM trial

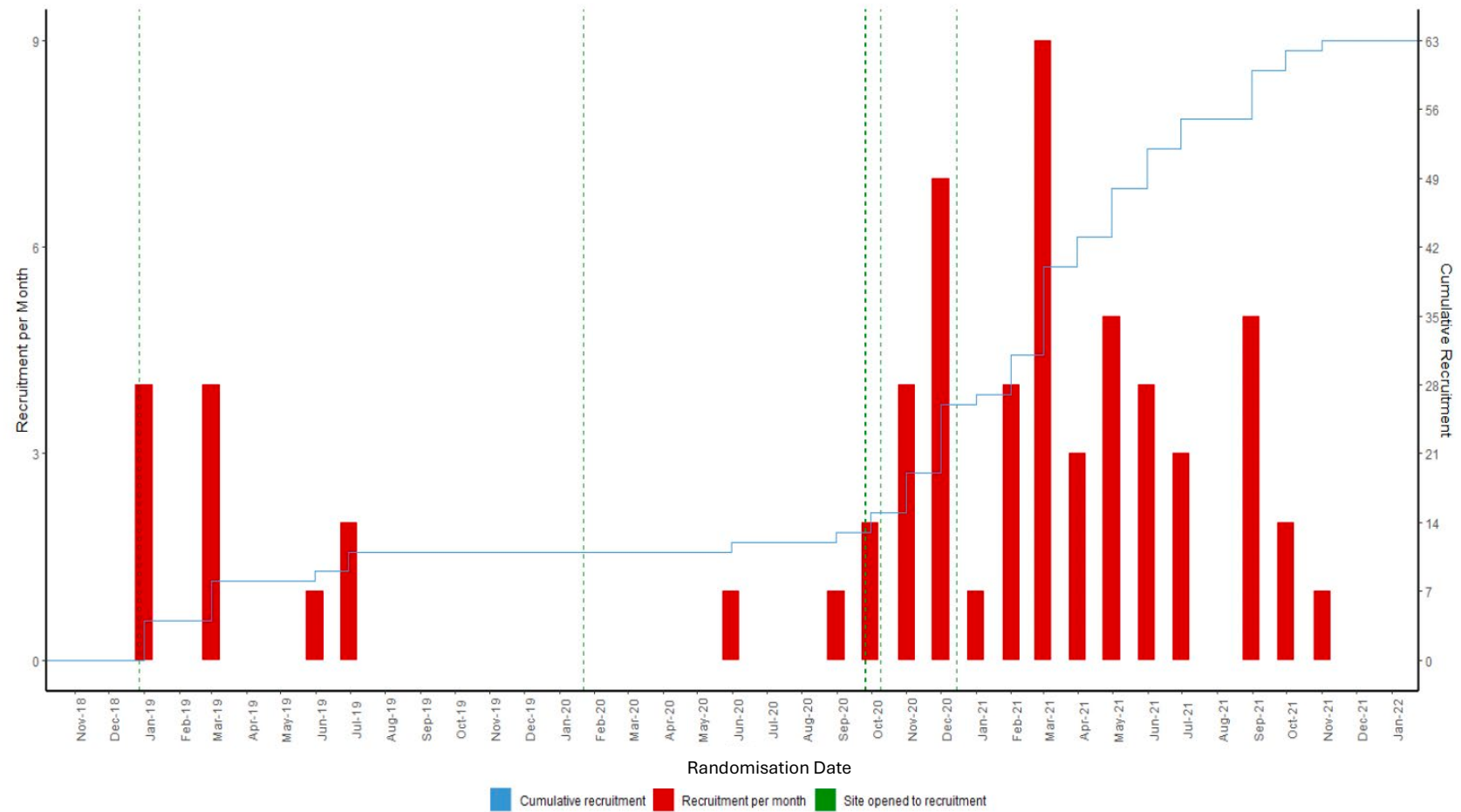

Supplement: online supplemental file 8 [file bmjopen-15-2-s008.pdf]
