# Supplementary material for: Sodium valproate, a potential repurposed treatment for the neurodegeneration in Wolfram syndrome (TREATWOLFRAM): trial protocol for a pivotal multicentre, randomised double-blind controlled trial
Source: BMJ Open. 2025 Feb 26;15(2):e091495. doi: 10.1136/bmjopen-2024-091495 (PMC11865774; doi:10.1136/bmjopen-2024-091495)
Supplement: online supplemental file 9 [file bmjopen-15-2-s009.pdf]

Supplementary appendix 9: Summary of TREATWOLFRAM trial protocol amendments

The following amendments and/or administrative changes have been made to this protocol since the implementation of the first approved version

Amendment number	Date of amendment	Protocol version number	Type of amendment	Summary of amendment
01	19-Sep-2018	3.0	Substantial amendment	Modification of contact details for the UK coordinating centre, clinical coordinators and European recruiting centre<br>Removal of exclusion criterion relating to POLG testing, and justification of removal<br>Clarification of exclusion criteria related to liver dysfunction<br>Modification of assessments schedule:<br>Patient questionnaires removed from visit 4<br>Pregnancy test added to visit 5<br>Tanned pubertal stage added at visit 1 and 11<br>Schedule of event amended to match the text – patient diaries collection/review and Mood questionnaires as per section 8.1, list of biochemistry tests as per section 7.3, sodium valproate listed as a separate line as per section 7.6 (treatment compliance).<br>Addition of Sleep questionnaires list<br>Clarification of optional tests/questionnaires for international sites<br>MRI safety recommendation added and date of first MRI extended from 90 to 180 days of visit 1.<br>Addition of imaging exploratory objectives and outcomes<br>Clarification of treatment administration schedule and procedure<br>Addition of ISRCTN reference number Addition of text relating to the GDPR<br>List of abbreviations updated<br>List of CRF updated<br>Commercial name of IMP changed from 'Orlept' to 'Sodium Valproate'<br>Typos corrected and minor clarifications throughout
02	04-Jul-2019	4.0	Substantial amendment	Modification of National coordinating centres details<br>Clarification of secondary outcomes and update to exploratory objectives and outcomes

Amendment number	Date of amendment	Protocol version number	Type of amendment	Summary of amendment
				Change to inclusion criteria related to Wolfram syndrome diagnosis, and to pregnancy test and for patients unable/unwilling to have an MRI scan<br>Modification of assessment schedule:<br>Removal of Smell test<br>Removal of skin biopsy at visit 7<br>Addition of Research blood samples collection at visit 6<br>Patient diaries no longer collected at Visit 3 and 5<br>Biochemistry tests to include urine osmolarity<br>Text amended throughout in accordance with Valproate pregnancy prevention programme and addition of links for each country (Appendix 5)<br>Pregnancy test amended for visit 1 (serum test preferred)<br>Section about allowing Share care (outside the UK) and Trial Steering Committee added<br>Clarification of treatment administration schedule, procedure and dosing<br>Additional recommendations for patients who discontinue IMP treatment and for patients who turn 12 while being on treatment.<br>Pharmacovigilance reporting and unblinding procedure updated<br>Update to lists of patient questionnaires, CRF and concomitant medication to be used with caution<br>Schedule of event amended to match the text – time range for visits changed<br>Addition of all contact numbers for ESMS (unblinding services)<br>Update to sections 16, 17 and 18.<br>List of abbreviations updated<br>Typos corrected and minor clarifications throughout
04	14-Nov-2019	4.0a	Substantial amendment	Addition of instructions to patients and research staff about the completion and review of Trial Medication Diaries and amendment to wording on Trial Medication Diaries.
01	14-Nov-2019	4.0a	Non-Substantial amendment	Modification of Spain Clinical coordinator address
05	19-Feb-2021	5.0	Substantial Amendment	Addition of Co-Investigators and Clinical coordinators<br>Change to Trial Statisticians<br>Change to minimum inclusion age (6 years old), addition of minimum inclusion weight (20kg)<br>Addition of Thyroid Function tests at visits 1, 5, 7, 9 and 11.

Amendment number	Date of amendment	Protocol version number	Type of amendment	Summary of amendment
				Additional methods for measurement of pancreatic beta cell reserve<br>Changes to schedule of events, including guidance in case of COVID-19 restrictions at site<br>Removal of carnitine and lactate dehydrogenase from biochemistry assessments<br>Addition of ICIQ-CLUTS caregiver form<br>Clarification to assessments that should be performed in case of early IMP discontinuation (face-to-face visit and telephone call), including collection of blood sample for Sodium valproate levels.<br>Guidance on central review of visual acuity data, including expedited review of inclusion data where required<br>Additional wording for assent, and consent of patients reaching the majority while being on trial<br>Addition of a secondary outcome (Contrast sensitivity, if available)<br>Clarification on method used for measuring colour vision (Hardy Rand and Rittler test)<br>Change to contact details for ESMS and CRCTU<br>Addition of dose increase/restart instructions after initial dose reduction/treatment discontinuation<br>Change to Safety lab abnormality IMP discontinuation<br>Addition of recommended questions for Telephone assessments<br>Guidance on questionnaire administration<br>Clarification of SAE reporting period<br>Clarification for AE reporting of abnormal blood results<br>Addition of wording previously required by France and Spain Competent Authorities<br>Addition of dosing table for patients reaching 12 years of age<br>List of abbreviations updated<br>Typos corrected and minor clarifications throughout
08	13-Sep-2022	6.0	Substantial Amendment	Change to Senior Statistician and Trial Coordinator.<br>Corrections to Amendments table.<br>Changes to the Investigational Medicinal Product Dossier (IMPD): Sharp Clinical Services UK Ltd to package, label and distribute IMP and Sharp Clinical Services UK Ltd to manufacture, package, label and distribute IMP-matched placebo.<br>Addition of dose reduction guidance for patients with renal impairment.

Amendment number	Date of amendment	Protocol version number	Type of amendment	Summary of amendment
				Serious Adverse Event (SAE) reporting updated: SAE form to be submitted by email and references to submission by fax removed.<br>Typos corrected.
09	12–Jan-2023	7.0	Substantial Amendment	Addition of a new section 7.5 - specifically in relation to dose interruptions due to any other reason which include IMP logistical delays/interruption in supply- at a local of study level<br>Non-substantial: change to trial management staff; Trial coordinator
10	19-Sep-2023	8.0	Substantial Amendment	Update to SmPC and Reference Safety Information (RSI). Addition of Metamizole to section 7.8.2.<br>Change to Senior Trial Statistician and Trial Coordinator.<br>Typographical errors corrected.
