# Supplementary material for: Sodium valproate, a potential repurposed treatment for the neurodegeneration in Wolfram syndrome (TREATWOLFRAM): trial protocol for a pivotal multicentre, randomised double-blind controlled trial
Source: BMJ Open. 2025 Feb 26;15(2):e091495. doi: 10.1136/bmjopen-2024-091495 (PMC11865774; doi:10.1136/bmjopen-2024-091495)
Supplement: Supplementary file 10 [file bmjopen-2024-091495.reviewer_comments.pdf]

PEER REVIEW HISTORY

BMJ Open publishes all reviews undertaken for accepted manuscripts. Reviewers are asked to complete a checklist review form and are provided with free text boxes to elaborate on their assessment. These free text comments are reproduced below.

ARTICLE DETAILS

Title (Provisional)

Sodium valproate, a potential repurposed treatment for the neurodegeneration in Wolfram syndrome (TREATWOLFRAM): trial protocol for a pivotal multi-centre, randomised double-blind controlled trial.

Authors

Dias, Renuka P; Brock, Kristian; Hu, Kun; Gupta, Rajat; Martin, Una; Peet, Andrew; Wilson, Martin; Yu-Wai-Man, Patrick; Wright, Benjamin; Mollan, Susan; Kulkarni, Archana; Meunier, Isabelle; Billingham, Lucinda; Nagy, Zsuzsanna; Rose, Heather; Koks, Sulev; Zatyka, Malgorzata; Astuti, Dewi; Lynch, Tracy; Morrison, Karen E.; Barton, Darren; Cronier, Sabrina; Malpass, Rebecca; Evans, Ruth; Malhi, Amandip; Takhar, Pooja; Lamb, Amy; Esteban-Bueno, Gema; Młynarski, Wojciech; Orssaud, Christophe; Roubertie, Agathe; Homer, Victoria; Barrett, Timothy

---

VERSION 1 - REVIEW

**Reviewer**	**1**
**Name**	**Serbis , Anastasios**
**Affiliation**	**University of Ioannina**
**Date**	**23-Aug-2024**
**COI**	**None**

I would like to thank Dias et al for this trial protocol. It is an appropriately designed protocol for a very rare progressive disease. The paper is well written with sufficiently described methods, and clearly defined outcomes. I only have the following minor suggestions:

1. It is true that the main features of WS1 are DM and optic atrophy. Nevertheless, DI and sensorineural hearing loss have been also considered important (hence the acronym DIDMOAD) and, indeed, appear in up to 2/3 of the patients and usually by the second decade of life. I would, therefore, suggest to "upgrade" these two clinical findings in the first paragraph of the Introduction section and to report them at the beginning of the paragraph ("Diagnosis requires symptoms of..." of as other complications.

2. Line 132: please rephrase the sentence "We therefore designed a trial with rate of vision loss as the primary outcome." Several other factors (including ease of follow up, frequency

of diagnosis and so on) played a role in your decision and not only what patients defined as important symptoms.

3. Line 208: Since this is the first point where you mention the possible side effects of VPA on a possible pregnancy, please elaborate more on the specific measures taken to avoid such a senario. For exampe, you can include the sentence "If sexually active, they must agree to use a highly effective contraception measure and to pregnancy testing at each clinic follow up visit" from Table 1.

Reviewer **2**

Name **Feil, Katharina**

Affiliation **Hertie-Institute for Clinical Brain Research, University of Tuebingen, Tuebingen, Germany.; Centre for Neurology, Department for Vascular Neurology, University of Tuebingen, Tuebingen, Germany**

Date **17-Sep-2024**

COI **I do not have competing interests.**

Thank you for the opportunity to review your manuscript. Overall, this is a comprehensive and well-prepared study protocol addressing a significant gap in the treatment of Wolfram syndrome. The inclusion of robust methodology and ethical considerations makes it a valuable contribution to the field, assuming the ongoing nature of the study is maintained.

Here are some considerations:

General Assessment

Study Design: The protocol outlines a well-structured, multicenter, double-blind, placebo-controlled, randomized clinical trial. It clearly states the primary and secondary objectives, inclusion and exclusion criteria, and detailed methodology.

Rationale and Background: The introduction provides a strong rationale for the study, including the unmet need for treatment in Wolfram syndrome and the hypothesis that sodium valproate may slow neurodegeneration. The background is comprehensive and makes a compelling case for the study.

Ethical Considerations: The protocol discusses ethics approval and participant consent processes, including special considerations for a vulnerable population (children with a rare disease).

Specific Points to Address

Study Dates: The manuscript indicates that recruitment started in January 2019 and will conclude in November 2024. It is crucial to ensure that this ongoing status is maintained

throughout the review process since completed data collection may render the protocol ineligible for publication in BMJ Open.

Inclusion and Exclusion Criteria: The criteria are well defined, including a focus on children above 6 years and adults with confirmed pathogenic mutations in the Wolfram gene. However, further justification for the exclusion of patients under six years or those with significant vision loss could be elaborated.

Randomization and Masking: The protocol appropriately details the randomization process (2:1 allocation in favor of VPA) and the steps taken to ensure blinding. It would be beneficial to confirm if any unforeseen unblinding has occurred and how it will be handled.

Outcome Measures:

Primary Outcome: The choice of visual acuity (VA) as the primary outcome is justified by patient and regulatory input. The method for measuring and analyzing VA is detailed.

Secondary Outcomes: A comprehensive list is provided, including safety, tolerability, MRI measures, and quality of life. This strengthens the protocol.

Statistical Analysis: The sample size calculation is based on robust methodology, considering the rare nature of the condition. However, only 63 of the planned 70 participants were recruited, a point that should be discussed in terms of its potential impact on study power and outcomes.

Challenges and Limitations:

COVID-19 Impact: The protocol appropriately addresses challenges due to the pandemic, including modifications for remote visits and home-based assessments. This adaptability is commendable, but the potential impact on data consistency should be acknowledged.

Generalizability: The study focuses on a very specific population (Wolfram syndrome patients) and may have limited generalizability. However, this is typical and expected for research on ultra-rare diseases.

Recommendations

Clarifications: Consider adding more detail about the rationale for excluding certain patient groups (e.g., children under 6 years).

Power and Sample Size: Discuss how the shortfall in recruitment (63 out of 70 participants) might affect the study's power and the interpretation of results.Eventually this could also be discussed in the main results paper.

Future Directions: Given the study's potential impact, a brief section on how positive results might influence future research or clinical practice could add value.

**Reviewer**	**3**
**Name**	**Saha, Lekha**

Affiliation **Department of Pharmacology, Post Graduate Institute of Medical Education & Research (PGIMER), Chandigarh 160012, India.**

Date **11-Dec-2024**

COI **None**

---

1. Regarding Masking, it is not very clear from the protocol how blinding was performed during COVID 19 please elaborate.

2. It is also not very clear from the protocol how participants were follow during COVID 19 please elaborate in the protocol.

3. Some of the references are very old like 1989 please replaced with recent one.

VERSION 1 - AUTHOR RESPONSE

Reviewer 1 Comments	Authors’ Response
I would like to thank Dias et al for this trial protocol. It is an appropriately designed protocol for a very rare progressive disease. The paper is well written with sufficiently described methods, and clearly defined outcomes. I only have the following minor suggestions:	Dear Dr Serbis,<br>Thank you for your kind comments and minor suggestions which we have endeavoured to answer below:
It is true that the main features of WS1 are DM and optic atrophy. Nevertheless, DI and sensorineural hearing loss have been also considered important (hence the acronym DIDMOAD) and, indeed, appear in up to 2/3 of the patients and usually by the second decade of life. I would, therefore, suggest to "upgrade" these two clinical findings in the first paragraph of the Introduction section and to report them at the beginning of the paragraph ("Diagnosis requires symptoms of..." of as other complications.	Thank you for this comment. The clinical diagnostic criteria for classic Wolfram-like spectrum disorder remain optic atrophy and diabetes mellitus diagnosed < 16 years. We have amended the introduction to make clear that DI, sensorineural deafness, and neurogenic bladder, occur in up to 2/3 of affected people.
Line 132: please rephrase the sentence "We therefore designed a trial with rate of vision loss as the primary outcome." Several other factors (including ease of follow up, frequency of diagnosis and so on) played a role in your decision and not only what patients defined as important symptoms.	Other factors included availability of natural history data on visual acuity; linear progression over time; a standardised and reproducible method of measurement; and ability to take repeated measurements over time. We have incorporated these: “In addition to being a patient relevant outcome measure, natural history data in visual

	acuity was available; visual acuity showed linear progression over time; there was a standardised and reproducible method of measurement; and it was possible to take repeated measures over time.”
Line 208: Since this is the first point where you mention the possible side effects of VPA on a possible pregnancy, please elaborate more on the specific measures taken to avoid such a senario. For exampe, you can include the sentence "If sexually active, they must agree to use a highly effective contraception measure and to pregnancy testing at each clinic follow up visit" from Table 1.	We have added the following: “The use of VPA in pregnancy is associated with a teratogenic risk to the foetus. Therefore an inclusion criterion was that all women of childbearing potential must use a highly effective method of contraception, without interruption, for the whole of the IMP period.”
Reviewer 2 comments	Authors’ response
Thank you for the opportunity to review your manuscript. Overall, this is a comprehensive and well-prepared study protocol addressing a significant gap in the treatment of Wolfram syndrome. The inclusion of robust methodology and ethical considerations makes it a valuable contribution to the field, assuming the ongoing nature of the study is maintained.	Dear Dr Feil,<br>Thank you for your generous comments, in particular about the robustness of the methodology and ethical considerations.
Study Design: The protocol outlines a well-structured, multicenter, double-blind, placebo-controlled, randomized clinical trial. It clearly states the primary and secondary objectives, inclusion and exclusion criteria, and detailed methodology.<br>Rationale and Background: The introduction provides a strong rationale for the study, including the unmet need for treatment in Wolfram syndrome and the hypothesis that sodium valproate may slow neurodegeneration. The background is comprehensive and makes a compelling case for the study.<br>Ethical Considerations: The protocol discusses ethics approval and participant consent processes, including	Thank you for your positive comments

special considerations for a vulnerable population (children with a rare disease).	
Study Dates: The manuscript indicates that recruitment started in January 2019 and will conclude in November 2024. It is crucial to ensure that this ongoing status is maintained throughout the review process since completed data collection may render the protocol ineligible for publication in BMJ Open.	Thank you. Recruitment has concluded. Data collection is ongoing and is anticipated to continue through to end of February 2025.
Inclusion and Exclusion Criteria: The criteria are well defined, including a focus on children above 6 years and adults with confirmed pathogenic mutations in the Wolfram gene. However, further justification for the exclusion of patients under six years or those with significant vision loss could be elaborated.	Exclusion of patients under 6 years:<br><br>We initially excluded patients under 5 years due to the unpredictability of being able to swallow VPA tablets, and the difficulty of matching a placebo to a liquid preparation. The age was then raised to 6 years due to regulatory issues in one of the participating countries.<br><br>Significant vision loss:<br><br>Most participants had significant visual loss. Patients whose vision loss was such that it could not be accurately quantified on a standard EDTRS chart, were not included in the study, as it would not be possible to measure further progression of that vision loss in response to IMP or placebo.<br><br>We have not added this to the main paper as these justifications are within the main Trial Protocol.
Randomization and Masking: The protocol appropriately details the randomization process (2:1 allocation in favor of VPA) and the steps taken to ensure blinding. It would be beneficial to confirm if any unforeseen unblinding has occurred and how it will be handled.	We have carefully monitored the pathway from randomisation, IMP/placebo pack distribution, dispensing, and return of unused tablets, at each participating site, and with site staff and study monitors. We are confident that no unforeseen unblinding has occurred.
Outcome Measures:<br>Primary Outcome: The choice of visual acuity (VA) as the primary outcome is justified by patient and regulatory input. The method for measuring and analyzing VA is detailed.<br>Secondary Outcomes: A comprehensive list is provided, including safety,	In relation to 63 participants being recruited rather than 70, we calculated the minimum number of participants required to achieve 80% power while retaining a minimal type 1 error. We were advised by the EMA protocol assistance committee that there could be some flexibility in the latter and that a type 1

tolerability, MRI measures, and quality of life. This strengthens the protocol.<br>Statistical Analysis: The sample size calculation is based on robust methodology, considering the rare nature of the condition. However, only 63 of the planned 70 participants were recruited, a point that should be discussed in terms of its potential impact on study power and outcomes.	error as large as 10% could be acceptable. Further work by our trial statistician showed that this could be achieved by recruiting a minimum of 63 participants while allowing up to 20% attrition. We have added the following text to reflect this: "We calculated the minimum number of participants required to achieve 80% power while retaining an acceptable type 1 error. Accepting a type 1 error of 10%, we should still be able to achieve 80% power with 63 participants, while allowing for up to 20% attrition."
Challenges and Limitations:<br>COVID-19 Impact: The protocol appropriately addresses challenges due to the pandemic, including modifications for remote visits and home-based assessments. This adaptability is commendable, but the potential impact on data consistency should be acknowledged.<br>Generalizability: The study focuses on a very specific population (Wolfram syndrome patients) and may have limited generalizability. However, this is typical and expected for research on ultra-rare diseases.	The primary outcome is visual loss and all these assessments were undertaken in hospital as planned as were MRI scans. Therefore we do not anticipate any impact on data consistency due to the COVID 19 pandemic.
Recommendations<br>Clarifications: Consider adding more detail about the rationale for excluding certain patient groups (e.g., children under 6 years).	Thank you. As explained above, we initially excluded patients under 5 years due to the unpredictability of being able to swallow VPA tablets, and the difficulty of matching a placebo to a liquid preparation. The age was then raised to 6 years due to regulatory issues in one of the participating countries.<br><br>We have not added this to the main paper as these justifications are within the main Trial Protocol.
Power and Sample Size: Discuss how the shortfall in recruitment (63 out of 70 participants) might affect the study's power and the interpretation of results.Eventually this could also be discussed in the main results paper.	Thank you. As explained above, we calculated the minimum number of participants required to achieve 80% power while retaining a minimal type 1 error. We were advised by the EMA protocol assistance committee that there

	could be some flexibility in the latter and that a type 1 error as large as 10% could be acceptable. Further work by our trial statistician showed that this could be achieved by recruiting a minimum of 63 participants while allowing up to 20% attrition. We have added the following text reflect this: “We calculated the minimum number of participants required to achieve 80% power while retaining an acceptable type 1 error. Accepting a type 1 error of 10%, we should still be able to achieve 80% power with 63 participants, while allowing for up to 20% attrition.”
Future Directions: Given the study's potential impact, a brief section on how positive results might influence future research or clinical practice could add value.	We have added a further section to the discussion: “Future directions for research and clinical practice”:<br><br>“This trial design will set the standard for future multi-centre pivotal trials in ultra-rare diseases. The work done to ensure MR imaging data compatibility from 6 international sites will provide a template for other international studies collecting neuroimaging data.<br>Biosamples collected from the UK cohort over the 36 months intervention will support investigations into biomarkers that may be used as surrogate outcome measures to shorten the intervention period in future studies.<br>Standardised natural history data from patients on placebo will be available to support future trial design using prior hypotheses and historical natural history data, thereby reducing participant numbers and intervention duration.<br>If the trial demonstrates efficacy of VPA in slowing progression of visual acuity loss, then our group will seek an industry partner to prepare marketing authorisation applications for licensing of VPA for the treatment of neurodegeneration in Wolfram syndrome. The clinical data collected will inform clinical teams and patients

	on clinical management and screening for complications. “
Reviewer 3 comments	Authors’ responses
Regarding Masking, it is not very clear from the protocol how blinding was performed during COVID 19 please elaborate.	Blinding was maintained throughout the study period including during the COVID 19 pandemic. IMP or placebo was dispensed from hospital clinical trials pharmacies, and couriered to patients in their homes. At no point were participants, or their hospital teams, able to access treatment allocations. This information was restricted to the trial statistician throughout the whole duration of the trial.
It is also not very clear from the protocol how participants were follow during COVID 19 please elaborate in the protocol.	Trial visits to hospital were maintained for visual acuity assessment and neuroimaging. During the Covid-19 pandemic, visits 3 and 5 were conducted remotely. Site investigators were trained to collect data on patient diaries, adverse events, and mood questionnaires, by telephone. This is detailed in the protocol.
Some of the references are very old like 1989 please replaced with recent one.	We thank the reviewer for this comment. We cannot alter this reference as this is the primary reference for the questionnaire used in the study protocol
